# FORCE: FORward modeling for Complex microstructure Estimation

**DOI:** 10.21203/rs.3.rs-8151109/v1

**Published:** 2025-11-20

**Authors:** Atharva Jaydeep Shah, Rafael Neto Henriques, Alonso Ramirez-Manzanares, Patryk Filipiak, Steven Baete, Kaustav Deka, Maharshi Gor, Serge Koudoro, Eleftherios Garyfallidis

**Affiliations:** 1 Indiana University, Bloomington, Indiana, USA.; 2 Instituto de Biofísica e Engenharia Biomédica, Faculdade de Ciências da Universidade de Lisboa, Lisbon, Portugal.; 3 Champalimaud Research, Champalimaud Foundation, Lisbon, Portugal.; 4 Centro de Investigación en Matemáticas A.C. (CIMAT), Guanajuato, Mexico.; 5 Center for Advanced Imaging Innovation and Research, NYU Langone Health, New York, USA.

**Keywords:** Diffusion-weighted MRI (dMRI), Fiber reconstruction, Simulation-based, Microstructure modeling, Forward modeling, Biophysics

## Abstract

Diffusion Magnetic Resonance Imaging (dMRI) is a noninvasive modality that enables the study of brain tissue microstructure and the reconstruction of neural pathways. To achieve this, most reconstruction methods rely on inverse modeling techniques, which are often ill-posed and struggle to resolve shallow fiber crossings. Moreover, existing methods typically focus either on estimating fiber orientations or on deriving microstructural maps. As a result, obtaining a comprehensive characterization of tissue microstructure and architecture often requires combining multiple models, which is computationally demanding, potentially inconsistent due to model-specific assumptions and acquisition settings. This work introduces FORCE, a forward modeling paradigm that reframes how diffusion data is analyzed. Instead of inverting the measured signal, FORCE simulates a large set of biologically plausible intra-voxel fiber configurations and tissue compositions. It then identifies the best-matching simulation for each voxel by operating directly in the signal space. This unified framework simultaneously resolves low-angle fiber crossings, producing a large suite of microstructural maps and complete tissue segmentation in a single process. The proposed approach demonstrates robust performance across synthetic and real datasets from both human and mouse brains, encompassing multiple resolutions and acquisition types.

## 1 Introduction

**Fig. 1:**
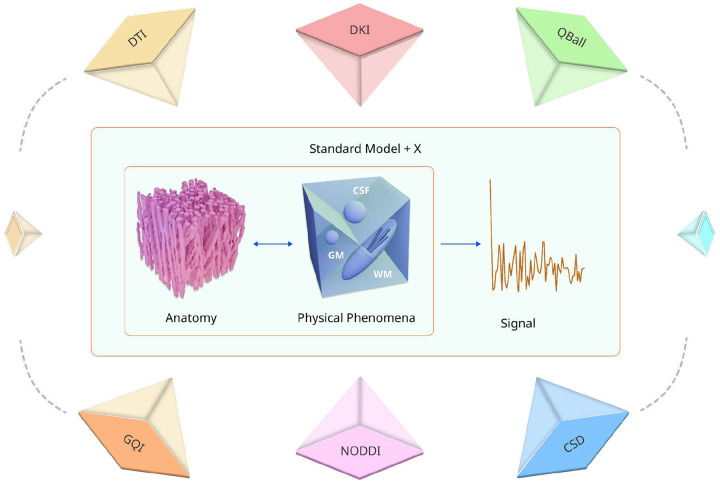
The diffusion-weighted signal encodes rich microstructural information. The standard diffusion model represents a simplified mixture of WM and Free Water (FW), describing signal attenuation within these tissue types. Extensions to this model, referred to as Standard Model + X, add components or priors (X) to capture more complex tissue features such as dispersion, Gray Matter (GM) fraction. Traditional inverse models apply specific constraints or priors such as Gaussian diffusion for Diffusion Tensor Imaging (DTI), non-Gaussian diffusion for Diffusional Kurtosis Imaging (DKI), fiber response for Constrained Spherical Deconvolution (CSD), spherical basis function for Q-Ball or compartmental representations for Neurite Orientation Dispersion and Density Imaging (NODDI) to recover select features. These inverse models can be viewed as lower dimensional projections of the same signature signal. However, this signal is simply put the outcome of a single generative biophysical model operating on the actual underlying anatomy.

Diffusion Magnetic Resonance Imaging (dMRI) has become an indispensable noninvasive tool in neuroscience and medical imaging, enabling researchers to investigate the brain’s microstructure and connectivity in vivo [1, 2]. It plays a critical role in understanding the relationship between early brain microstructural alterations and its function, studying neurological disorders, and mapping structural connectivity across individuals and populations [3–5]. Diffusion MRI quantifies the random motion of water molecules within brain tissue by measuring signal attenuation along multiple noncollinear diffusion gradients [6]. Because this molecular motion is affected by the microstructural organization of the tissue, the diffusion-weighted signal varies depending on the direction and strength of the applied gradients. These directional dependencies form the foundation for identifying White Matter (WM) tracts and conducting other dMRI-based analyses [7, 8]. A large literature has focused on understanding the biophysical underpinnings of dMRI and linking microstructure to the measured diffusion signal [9–11]. Regardless of framework, the inference is performed on the same diffusion-weighted signal, with differences arising from modeling choices rather than distinct data [12, 13]. Figure 1 illustrates how diffusion physics links microstructural anatomy to the measured signal and how different methods transform this mapping to derive microstructural maps. Thus, much of the relevant microstructural information is already encoded in the acquired diffusion-weighted signal.

For decades, the field has relied on a variety of inverse modeling techniques to decode this information. Among them, Diffusion Tensor Imaging (DTI) is a widely used model that identifies primary direction of diffusion and produces quantitative maps such as Mean, Axial, and Radial Diffusivities (MD, AD, RD), and Fractional Anisotropy (FA) [1]. This technique has been used to assess various neurological conditions and brain functions and connectivity [2, 3, 14, 15]. Numerous articles have evaluated neurological conditions based on DTI-based fiber tractography and metrics, but a known limitation of DTI is that it cannot resolve complex fiber crossings in WM pathways and its diffusion parametric maps are sensitive to confounding variables [4, 5, 12, 13]. As an extension to DTI, Diffusional Kurtosis Imaging (DKI) was introduced to capture the non-Gaussian behavior of water diffusion, providing additional metrics such as Mean, Axial, Radial Kurtosis (MK, AK, RK) and Kurtosis Fractional Anisotropy (KFA) [16–18].

On the other hand, advanced methods have been proposed to resolve the fiber crossings, including Q-ball imaging and its variants like Constant Solid Angle (CSA)[13, 19, 20], Constrained Spherical Deconvolution (CSD) family of models (Single-Shell Single Tissue-CSD, Multi Shell Multi Tissue-CSD, Single-Shell 3 Tissue-CSD) [21–23], Generalized Q-sampling Imaging (GQI) [24] among many others. Q-ball imaging, the CSD family, and GQI each have a microstructure summary metric, specifically Generalized Fractional Anisotropy (GFA), Apparent Fiber Density (AFD), and Quantitative Anisotropy (QA), respectively. These techniques operate by transforming the diffusion signal from its native measurement space into an Orientation Distribution Function (ODF) typically through mathematical transformations such as spherical harmonics decomposition or deconvolution using an estimated response function. These methods have shown effectiveness in resolving multiple fiber orientations in a voxel but often fail to resolve crossing fibers at angles typically below 40° due to limited acquisition and ODF peak width [25, 26]. This limitation also affects the downstream task of tractography, as most tractography algorithms rely on detecting local maxima of the ODFs to infer fiber pathways, potentially leading to erroneous or incomplete reconstructions in regions of complex fiber crossings. Moreover, these models often do not yield directly interpretable microstructural parameters, such as the volume fraction, diffusion anisotropy, or kurtosis of individual tissue compartments limiting their interpretability and biological relevance in clinical or research settings [27]. These limits of ODF-based methods motivate an approach that does not rely on deconvolution or finding maxima of ODF for peaks.

These limitations have also motivated the exploration of machine learning techniques, especially deep learning, which aim to learn fiber orientations or ODFs directly from the measured signals. These methods often train neural networks to directly regress fiber orientations or estimate ODFs from diffusion-weighted signals [28–31]. However, such approaches typically require large, high quality training datasets and careful tuning to generalize well. Even under idealized or well-curated training conditions, deep learning models for fiber reconstruction rarely generalize reliably. In many cases, their performance suffers significantly when the training data distribution does not perfectly match the test conditions, limiting their robustness and clinical applicability [32]. Also, the lack of interpretability, as well as sensitivity to noise and acquisition variability, remain important concerns. Moreover, diffusion MRI operates in a symmetric domain, where multiple underlying tissue configurations may lead to the same observed signal, limiting the uniqueness of model estimates. Deep learning models may misinterpret this symmetry and the inherent ambiguities, leading to hallucinated peaks or spurious fiber orientations not present in the true underlying tissue structure [33].

In parallel, microstructure-focused inverse techniques such as NODDI (Neurite Orientation Dispersion and Density Imaging), Bingham-NODDI, Accelerated Microstructure Imaging via Convex Optimization (AMICO) etc. have been proposed to estimate interpretable microstructure properties such as Neurite Density (ND), Orientation Dispersion Index (ODI) and Free Water (FW) fractions [34–36]. These approaches infer tissue properties by fitting multi-compartment biophysical representations of the diffusion signal. Both NODDI and Bingham-NODDI typically require multi-shell data and nonlinear optimization [34, 35]. AMICO simplifies these models by expressing them in a linear form, enabling faster and more robust parameter estimation. However, most of these models rely on fixed diffusivity assumptions and assume a single fiber population per voxel to stabilize optimization and avoid parameter degeneracy. A substantial body of work has also advanced biophysical modeling, including CHARMED [37] and AxCaliber [38] for restricted intra-axonal diffusion, VERDICT for tumor microstructure [39], and soma-/exchange-aware frameworks such as SANDI/SANDIX [40, 41] and NEXI [42]. While they offer valuable microstructural metrics, they do not explicitly estimate fiber orientations and are therefore less commonly used for tractography-based analyses.

This work challenges the current paradigm by advocating a move away from inverse modeling towards forward modeling, a transformative approach that has revolutionized other scientific disciplines by modeling complex behaviors directly from foundational physical principles and observed data. In neuroscience, forward modeling frameworks for Electroencephalography (EEG), functional MRI (fMRI) have transformed how neural activity is linked to measured signals [43, 44]. In the realm of materials chemistry, forward modeling has advanced discovery of new materials allowing researchers to reliably simulate and optimize material properties before synthesizing, leading to accelerated material discovery [45]. Seismic exploration and weather prediction underscore the power of forward modeling, as global weather forecasting based on numerical simulations constrained by physical laws, routinely outperforms traditional methods and is extremely reliable by leveraging physical constraints and simulating atmospheric patterns [46–48]. These successes motivate a forward approach in dMRI that replaces ill-posed inverse fits with a direct, biophysically grounded signal match.

In dMRI, forward modeling provides a powerful way to move beyond simple signal fitting by explicitly linking tissue microstructure to the measured diffusion signal. Simulation-based approaches exemplify this idea. For example, ODF-Fingerprinting (ODFFP) constructs a large number of signal simulations with a multi-compartment model [11] and associating them with ODFs obtained from a prior reconstruction such as GQI [49, 50]. Matching proceeds by comparing voxel ODFs, typically after a rotational alignment step that reduces the number of simulations but increases computational cost. In addition, because the signal model does not explicitly model fiber dispersion, microstructural interpretability can be limited. These considerations motivate an approach that estimates fiber orientations directly in signal space, incorporates dispersion, resolves shallow crossings, and remains computationally efficient across diverse acquisition protocols.

**Fig. 2:**
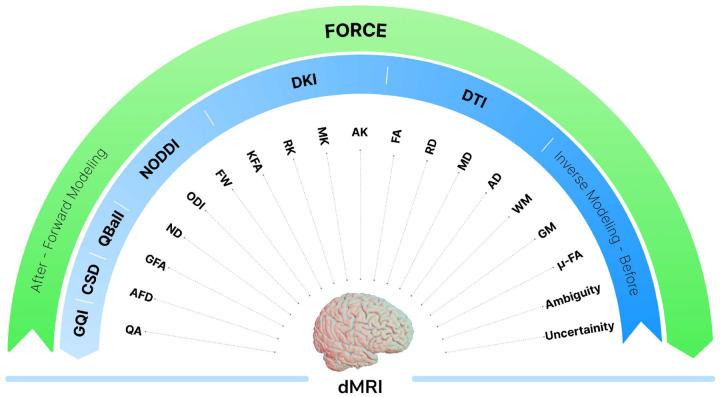
Traditional diffusion models rely on inverse modeling, where different models (e.g., DTI, NODDI, CSD) estimate specific microstructural properties. In contrast, FORCE adopts a forward modeling approach, generating biologically plausible signals that allow simultaneous estimation of fiber orientations and rich microstructure maps. The green arrow represents forward modeling from microstructure to diffusion-weighted signal, whereas the blue arrow represents inverse modeling from diffusion-weighted signal to microstructure. A single forward fit yields all maps, without inverse modeling.

This work introduces FORCE, a framework that embodies this forward modeling approach that aims to bridge the gaps mentioned above. By operating directly in the native signal space, FORCE sidesteps the entire signal-to-ODF process. It works by generating realistic, multi-compartment diffusion signals and identifying the best match for each voxel using a similarity metric. This enables high-resolution orientation reconstruction down to low crossing angles, while simultaneously providing voxel-wise microstructure estimates without requiring complex model fitting or optimization. Whereas conventional models such as DTI, NODDI, or DKI each recover only a subset of diffusion properties from the measured signal, FORCE can recover all such microstructural maps from a single unified framework. Figure 2 summarizes how the proposed framework differs from traditional inverse modeling approaches. The proposed approach is evaluated on synthetic and real dMRI datasets spanning multiple acquisition types and species, demonstrating higher reconstruction accuracy than state-of-the-art methods and providing comprehensive microstructural mapping.

## 2 Methods

### 2.1 Overview of the framework

FORCE generates biologically plausible diffusion-weighted signals and interprets the acquired data directly in signal space. For each voxel, the measured diffusion profile is compared to simulated signals using a penalized cosine similarity metric. The simulation with the highest similarity identifies the most likely microstructural configuration, from which fiber orientations, tissue composition, and scalar diffusion metrics are estimated.

### 2.2 Generation of synthetic signals

A large set of diffusion-weighted signals were simulated, each representing a unique microstructural configuration drawn from biologically plausible parameter ranges (Table 1). To assess the stability of the simulations, we evaluated the convergence of key DTI metrics (FA, MD, RD) and NODDI metrics (ODI, NDI, FW) as a function of simulation size (Figures C6, C7). The results show that FORCE estimates converge as the number of simulation grows, with negligible differences between 500 K and 1 M entries, indicating that the sampling density used in this work sufficiently captures the variability of the signal space. Although the simulation size can be increased for denser sampling, all analyses in this study use 500000 simulations unless explicitly stated otherwise. Each voxel is modeled as a mixture of WM, Gray Matter (GM), and FW, with WM containing up to three fiber populations. The simulation values follow the design principles outlined in prior work [50].

#### 2.2.1 Biophysical model

Each WM fiber population i is represented by an orientation distribution $p_{i}(n)$ (Bingham), intra and extra-axonal volume fractions $\left(f_{\text{in},i},f_{\text{ex},i}\right)$, and associated diffusivities. The overall signal is modeled as the weighted sum of WM, GM, and FW contributions, reflecting their respective partial volumes within each voxel.

##### Signal expression

For gradient direction g and b-value b, the signal is

(1) $$S=S_{0}\left[\sum_{i=1}^{N}\left(f_{\text{in},i}\int_{S^{2}}p_{i}(n)S_{\text{in}}(b,g,n)dn+f_{\text{ex},i}\int_{S^{2}}p_{i}(n)S_{\text{ex}}(b,g,n)dn\right)+f_{\text{FW}}S_{\text{FW}}(b)+f_{\text{GM}}S_{\text{GM}}(b)\right]$$

subject to

$$\sum_{i=1}^{N}\left(f_{\text{in},i}+f_{\text{ex},i}\right)+f_{\text{FW}}+f_{\text{GM}}=1$$

##### Component signal models

###### Intra-axonal (stick).

Water inside axons is assumed to have negligible radial diffusivity over the diffusion times used, so displacement is constrained along the local fiber axis n. The signal for gradient g and b-value b is

(2) $$S_{\text{in}}(b,g,n)=\text{exp}\left(-bD_{\|}(g\cdot n{)}^{2}\right)$$

where $D_{\|}$ is the axial intra-axonal diffusivity. The absence of a transverse term reflects the stick limit and captures strongly restricted radial motion.

###### Extra-axonal (zeppelin).

Water in the extra-axonal space exhibits hindered diffusion with anisotropy induced by the local fiber orientation. The diffusivity is greater along the fiber axis than perpendicular to it. The signal for gradient g and b-value b is

(3) $$S_{\text{ex}}(b,g,n)=\text{exp}\left(-b\left[D_{⊥}^{\text{ex}}+\left(D_{\|}^{\text{ex}}-D_{⊥}^{\text{ex}}\right)(g\cdot n{)}^{2}\right]\right)$$

where $D_{\|}^{\text{ex}}$ is the axial extra-axonal diffusivity and $D_{⊥}^{\text{ex}}$ is the radial extra-axonal diffusivity. This model captures the hindered but unrestricted diffusion in the extracellular space surrounding axons.

###### Orientation distribution (Bingham).

The distribution of fiber orientations within a voxel is modeled using the Bingham distribution on the unit sphere. For fiber population i, the probability density of orientation n is

(4) $$p_{i}(n)=\frac{1}{c\left(Z_{i}\right)}\text{exp}\left(n^{⊤}Z_{i}n\right)$$

where $Z_{i}$ is a concentration matrix that determines the dispersion and principal directions of the distribution, and $c\left(Z_{i}\right)$ is the normalization constant. This allows for modeling of crossing fibers and fiber dispersion.

###### Free water.

Isotropic diffusion of unbound water in cerebrospinal fluid or edema exhibits unrestricted Gaussian diffusion with no directional preference. The signal for b-value b is

(5) $$S_{\text{FW}}(b)=\text{exp}\left(-bD_{\text{FW}}\right)$$

where $D_{\text{FW}}$ is the free water diffusivity. This compartment is orientation-independent.

###### Gray matter.

Tissue in cortical and subcortical gray matter exhibits isotropic diffusion due to the lack of coherent fiber orientation. The signal for b-value b is

(6) $$S_{\text{GM}}(b)=\text{exp}\left(-bD_{\text{GM}}\right)$$

where $D_{\text{GM}}$ is the gray matter diffusivity, typically lower than free water due to the presence of cell membranes and organelles that hinder diffusion. This compartment is orientation-independent.

#### 2.2.2 Sampling priors and choices

Orientations were sampled uniformly over a unit sphere using a 724-vertex electrostatic grid, providing an angular resolution of approximately 4.1° between nearest vertices [51]. All parameter’s ranges and distributions used for the signal simulations are summarized in Table 1. The values were chosen to reflect biologically plausible microstructural properties reported in previous studies [11, 49, 52–56].

### 2.3 Signal matching and cosine similarity

Following signal synthesis, we perform voxel-wise matching by selecting, for each voxel, the simulated diffusion-weighted signal that is most similar to the measured DWI signal. For simulated signals of size L ${\left\{S_{i}\right\}}_{i=1}^{L}$, we compute cosine similarity,

(7) $$C_{i}=\text{cos}\left(S_{\text{voxel}},S_{i}\right)=\frac{S_{\text{voxel}}^{⊤}S_{i}}{\left\|S_{\text{voxel}}\right\|\left\|S_{i}\right\|},i=1,\ldots ,L$$

We then rank simulations and retain the top K=50 to form a local candidate set 𝒩,

(8) $$\pi =\underset{↓}{\text{argsort}}(C),\;𝒩=\left\{\pi_{1},\ldots ,\pi_{50}\right\}$$

To discourage overly complex configurations, we apply a penalty on the number of fiber populations and select

(9) $$\overset{ˆ}{i}=\underset{i\in 𝒩}{\text{arg max}}\left[\text{cos}\left(S_{\text{voxel}},S_{i}\right)-\alpha \;n_{\text{fibers}}^{\left(i\right)}\right]$$

In Equation 9, $n_{\text{fibers}}^{\left(i\right)}$ is the number of fiber populations in entry i, and α controls the penalty strength. With K=50,𝒩 typically contains plausible one, two, and three-fiber candidates; if only a single fiber count is represented, the comparison reduces to that class. This regularized selection balances sensitivity to true crossings with suppression of spurious peaks [50].

This choice of cosine similarity is further motivated by the noise and scaling characteristics of dMRI. Although dMRI noise is often Rician, standard preprocessing attenuates floor effects so that, in expectation across gradient directions, residuals are approximately centered. Under this weak assumption, projecting the voxel signal onto the matched simulation vector aggregates directional information, reducing the effects of noise in high dimensional representations [57, 58]. In addition, the non–diffusion-weighted (b=0) image should exhibit the highest intensity because diffusion gradients only attenuate signal; however, in some datasets (e.g., minimally preprocessed HCP), b=0 volumes can appear darker than diffusion-weighted images due to scanner drift, gradient nonlinearities, incomplete steady state, or imperfect preprocessing. Under such conditions, $S_{0}$-based normalization is unstable and may further amplify noise, motivating a scale-invariant, direction-focused similarity metric. Representing the diffusion MRI measurement as an N-dimensional vector, cosine similarity compares the direction (shape) of measured and simulated profiles independent of global scale and remains discriminative in high dimensions, whereas $\ell_{2}$ distance becomes less informative as dimensionality grows [59–61].

To formalize this comparison, let $y,d\in R^{n}$ denote the measured and predicted signal vectors respectively. The unscaled squared $\ell_{2}$ loss is:

(10) $$\|y-d{\|}^{2}=\|y{\|}^{2}+\|d{\|}^{2}-2y^{⊤}d$$

If either vector is multiplied by a scaling factor γ>0,

(11) $$\|\gamma y-d{\|}^{2}=\gamma^{2}\|y{\|}^{2}+\|d{\|}^{2}-2\gamma y^{⊤}d,$$

which is a convex quadratic in γ. Hence small scale mismatches dominate the objective regardless of the directional agreement between y and $d;\ell_{2}$ is *not* comparable across voxels with different scales. By contrast, cosine similarity:

(12) $$\text{cos}(y,d)=\frac{y^{⊤}d}{\left\|y\|\|d\right\|}\;\text{satisfies}\;\text{cos}(\gamma y,d)=\text{cos}(y,d)(\gamma >0),$$

i.e., it is scale invariant and depends only on the direction of the signal vectors. Moreover, even the *best* $\ell_{2}$ fit after optimizing a scale β for the prediction,

(13) $$\underset{\beta >0}{\text{min}}\|y-\beta d{\|}^{2}=\|y{\|}^{2}-\frac{{\left(y^{⊤}d\right)}^{2}}{\|d{\|}^{2}}=\|y{\|}^{2}\left(1-{\text{cos}}^{2}(y,d)\right),$$

reduces to a function of cosine but is still weighted by the *magnitude* $\|y{\|}^{2}$. Thus, without explicit scaling, $\ell_{2}$ over-weights high-intensity measurements and is driven by arbitrary scale, whereas cosine yields a clean, scale-invariant comparison of signal vectors. Figure C15 illustrates this phenomenon with a simple experiment: $\ell_{2}$ incorrectly matches a high-amplitude signal to a flat intermediate-intensity profile, while cosine correctly identifies the scaled signal with matching shape.

Conventional approaches often fit multi-exponential or multi-compartment models to the measured data, but such formulations can be numerically unstable and yield degenerate parameter spaces. For example, in NODDI, diffusivity values are fixed to mitigate these issues [34, 62]. Bayesian inference methods such as Markov Chain Monte Carlo (MCMC) can estimate parameters with uncertainty quantification but are computationally intensive and sensitive to noise, model selection, and prior specification. Posterior distributions become diffuse or multi-modal when decay constants overlap or amplitudes are unbalanced, and convergence requires extensive tuning and monitoring [63, 64].

In contrast, our simulation-driven framework avoids explicit parameter fitting altogether. By constructing biologically plausible signal profiles and identifying the most similar entry via cosine matching, it yields robust fiber-orientation and microstructural estimates without the instability or computational burden of traditional inference. This makes the method scalable, tractable, and well suited for whole-brain diffusion MRI applications.

### 2.4 Output Generation

Once the best-matching simulation is identified for each voxel, all associated properties from that entry are retrieved to generate the final outputs. The peak directions, voxel-wise scalar microstructural maps, including FA, MD, NDI, ODI and other metrics are directly retrieved from the matched configuration. Diffusion and kurtosis tensors, along with corresponding DTI and DKI scalar maps such as FA, MD, MK, AK, RK, and KFA, can be derived analytically from the multi-compartment mixture by following the DKI framework implemented in Diffusion Imaging in Python (DIPY) (Appendix A). In addition, the partial volume fractions assigned during simulation are used to produce fuzzy tissue segmentations of WM, GM, and FW that is cerebrospinal fluid (CSF).

### 2.5 Quantifying uniqueness and degeneracy

Instead of suppressing degeneracies in the signal space, FORCE explicitly characterizes them through two voxel-wise measures computed from the similarity profile of the top-50 nearest simulations: uncertainty and ambiguity. Let $s^{\left(v\right)}={\left\{s_{i}^{\left(v\right)}\right\}}_{i=1}^{K}$ denote the penalized cosine similarities within the local neighborhood ${𝒩}_{K}$ for voxel v, where K=50 and each $s_{i}=\text{cos}\left(S_{\text{voxel}},S_{i}\right)-\alpha \;n_{\text{fibers}}^{\left(i\right)}$ as in Equation 9.

#### Uncertainty.

Uncertainty is defined as the interquartile range (IQR) of the similarity profile:

(14) $$U(v)=P_{75}\left(s^{\left(v\right)}\right)-P_{25}\left(s^{\left(v\right)}\right),$$

where $P_{q}$ denotes the qth percentile across the K neighbors. A larger U(v) indicates a broader spread of plausible matches and thus reduced confidence in a unique solution.

#### Ambiguity.

Ambiguity reflects the width of the similarity peak at half its maximum (normalized by K):

(15) $$A(v)=\frac{1}{K}\sum_{i=1}^{K}⊮\left[s_{i}^{\left(v\right)}>\frac{1}{2}\underset{j}{\text{max}}\;s_{j}^{\left(v\right)}\right],$$

where ⊮[⋅] is the indicator function that equals 1 if the condition is true and 0 otherwise. A(v) thus approximates a normalized full width at half maximum (FWHM), with values near zero corresponding to a sharp, unique match and larger values indicating multiple near-degenerate solutions. The above definitions follow standard usage in statistics and signal processing [65]. Intuitively, uncertainty captures spread among near-matches, whereas ambiguity captures whether multiple matches are nearly tied. Together, these metrics provide an interpretable view of model degeneracy and, when their patterns shift across a sample in a cohort, can potentially serve as naive data-quality indicators as well.

## 3 Evaluation and Results

### 3.1 Synthetic Data Experiments

Two complementary synthetic experiments evaluated angular resolution and fiber-number estimation for FORCE against CSA, CSD, GQI, and ODFFP. We simulated 8000 two-fiber crossings equally distributed across eight angle bins (10° – 20° through 80°–90°) at $b=2000\;\text{s}/{\text{mm}}^{2}$ with 150 directions, added dispersion (ODI 0.01–0.30) and Rician noise (SNR 50, 20, 10). For inverse methods, peaks on the ODFs were detected using local maxima search with a 10° minimum separation and a 0.6 relative-amplitude threshold parameters using DIPY. Angular performance was quantified by the angular error and by the proportion of correctly resolved crossings within a 20° tolerance, following common practice [50].

For angular resolution, FORCE achieved the highest and most uniform peak-detection rates over angle bins, with clear advantages at shallow crossings (10°−40°). The $\alpha =10^{-5}$ setting provided the best over-all balance across angles and noise levels. Methods exhibiting stable peak detection across angle bins also exhibited the highest angular accuracy.

**Fig. 3:**
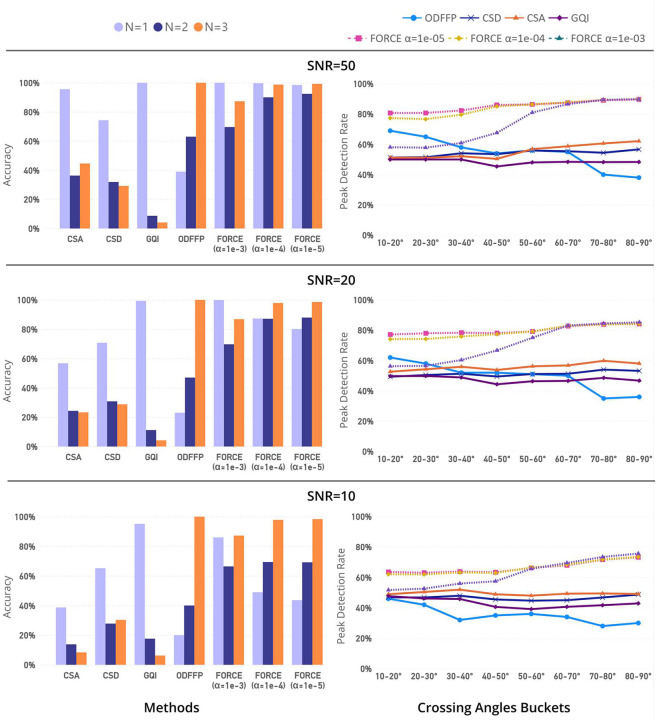
Left: Comparison of fiber number estimation accuracy across methods (CSA, CSD, GQI, ODFFP, FORCE) at different SNR levels (50, 20, 10) with dispersion. The accuracy (higher is better) is calculated based on correct identification of 1, 2, or 3 fibers from 10,000 synthetic configurations. Right: Comparison of fiber resolution (crossing-angle) estimation accuracy across methods (CSA, CSD, GQI, ODFFP, FORCE) at different SNR levels (50, 20, 10) with dispersion. The accuracy (higher is better) is calculated based on correct identification of angle simulated within 20° of error tolerance.

For fiber-number estimation, at high SNR all methods were accurate for single-fiber voxels. For multi-fiber voxels, FORCE yielded the highest accuracy and the least degradation as SNR decreased (Figure 3). GQI predominantly reported a single fiber across conditions, underestimating two and three-fiber cases, whereas ODFFP predominantly reported three fibers, overestimating the count even for single and two-fiber ground truth. CSA and CSD showed intermediate performance, with accuracy decreasing at smaller angles and lower SNRs. Among FORCE variants, $\alpha =10^{-5}$ achieved the best overall accuracy across SNRs and angle bins.

### 3.2 Phantom Data

Further validation was performed on the DiSCo (Diffusion Simulation and Construction) phantom [66], which provides a controlled environment with known fiber configurations. Methods were evaluated against the ground truth connectivity matrix using Pearson correlation, along with comparisons to current state-of-the-art approaches like CSD and MSMT-CSD. Since FORCE directly estimates fiber peaks, tractography was performed using the Euler Delta Crossings (EuDX) algorithm implementation in DIPY [67, 68]. On the DiSCo phantom at SNR levels of 50 and 10, FORCE produced orientation estimates that closely matched the ground truth and yielded connectivity matrices with high correlation to the reference reconstructions. For single-shell data with a b-value of 2000 s/mm^2^, FORCE achieved a correlation of 0.868 at SNR 10 (compared to 0.847 for CSD). At SNR 50, the correlation was 0.894 for FORCE compared with 0.902 for CSD. When compared with MSMT-CSD, FORCE achieved a correlation of 0.871 at SNR 10 versus 0.858 for MSMT-CSD, and 0.895 at SNR 50 versus 0.903 for MSMT-CSD when excluding the highest b-shell $\left(b=13000\;\text{s}/{\text{mm}}^{2}\right)$. This exclusion was performed because at such high diffusion weightings, the signal becomes extremely attenuated and difficult to interpret reliably using biophysical models, which can bias the comparison.

Minor adjustments were introduced to align the forward model with the characteristics of this numerical phantom as it departs from the regime of usual biological tissue diffusion parameters. To ensure consistency with the DiSCo simulation model, which represents diffusion using stick-like compartments, diffusivities were sampled from narrow uniform bands: $D_{\|}$ sampled from $\text{Uniform}(0.54,0.66)\times 10^{-3}{\text{mm}}^{2}/\text{s}$ and $D_{⊥}$ sampled from $\text{Uniform}(0.32,0.38)\times 10^{-3}{\text{mm}}^{2}/\text{s}$. The isotropic compartment was disabled to match the stick-like DiSCo model.

### 3.3 In vivo Diffusion Data

To qualitatively assess the applicability of the method to in vivo data, datasets spanning a range of spatial resolutions and diffusion weightings were processed:

- **Stanford HARDI dataset** [69]: Single-shell acquisition with $b=2000\;\text{s}/{\text{mm}}^{2}$ and 150 gradient directions; voxel size of $2\times 2\times 2\;{\text{mm}}^{3}$.
- **Human Connectome Project (HCP) 3T dataset** [70]: Multi-shell acquisition with b=1000,2000, and $3000\;\text{s}/{\text{mm}}^{2}$; 90 gradient directions per shell; voxel size of $1.25\times 1.25\times 1.25\;{\text{mm}}^{3}$.

Together, these datasets capture substantial variability in b-value strengths, voxel sizes, and SNRs, providing a comprehensive benchmark for real-world performance evaluation. Common parameters were used across datasets except a lower WM threshold (0.3 vs. 0.5) for Stanford HARDI due to larger voxels.

**Fig. 4:**
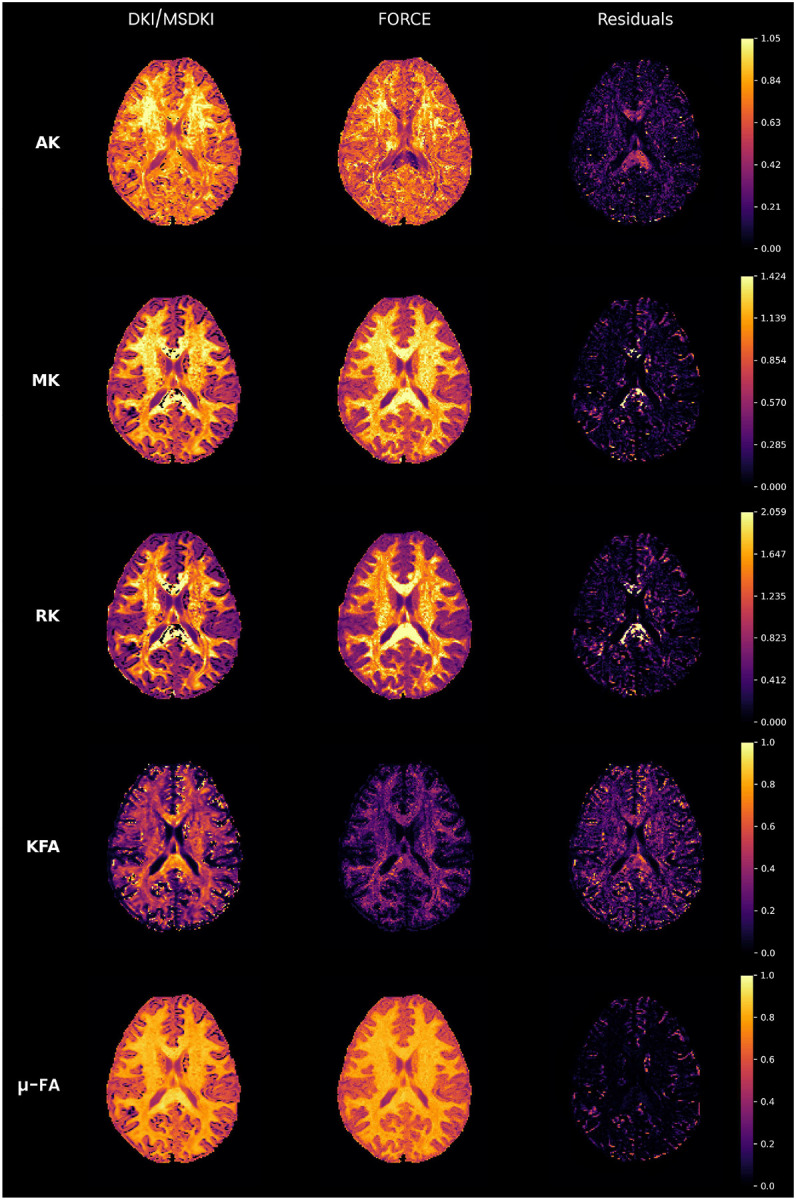
Comparison of DKI/Mean Signal DKI (MSDKI) and FORCE-derived parameter maps from the HCP dataset. Columns show DKI estimates (left), FORCE estimates (middle), and voxel-wise residuals (right) for AK, MK, RK, KFA. MSDKI estimates micro-FA. The same biomarkers are derived directly from FORCE (second column).

The evaluation focused on comparing microstructural indices derived from FORCE with those obtained using conventional model-fitting techniques on the HCP 3T dataset. As shown in Figure C1, FORCE exhibits high fidelity in reconstructing standard DTI metrics. The FA, MD, and RD maps are visually identical to those from standard DTI fitting, with minimal residuals, confirming that the method accurately preserves fundamental diffusion properties. The small residuals likely arise for three reasons. First, FORCE matches each voxel to the closest noiseless signal among the simulations, so measurement noise manifests as a residual even for the best match, while DTI fitting models the entire signal profile and absorbs measurement noise into its parameter estimates. Second, the complexity penalty in Equation 9 can steer the selection away from the unpenalized nearest neighbor, trading a small increase in misfit for reduced overfitting. Third, the simulations provide a discrete sampling of orientations and microstructural parameters, so an exact match may not exist. The advantages of FORCE become more evident in the higher order representations of DKI. In particular, the RK map, which is typically unstable in conventional fitting, appears smooth and anatomically consistent when estimated with FORCE, mitigating degeneracies in regions of complex fiber architecture (Figure 4, third row). Notably, the AK map generated by FORCE displays lower values in highly organized white matter tracts where FA is high, which reflects the expected neuroanatomical relationship (Figure 4, top row)[71].

**Fig. 5:**
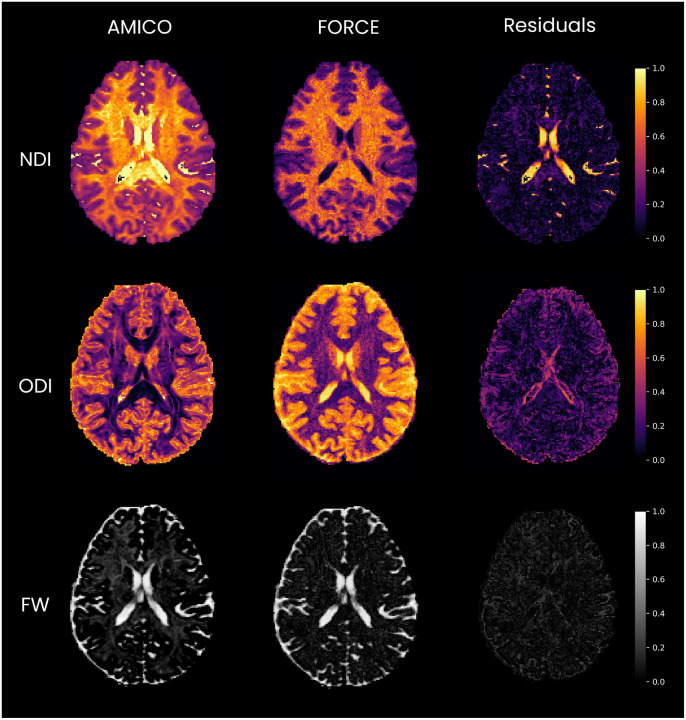
Comparison of NODDI parameter maps from AMICO (left) and FORCE (middle) on the HCP 3T dataset. The difference maps are shown on the right. FORCE produces sharper NDI and ODI maps with fewer artifacts. Notably, FORCE corrects for high ND values in the CSF observed in the AMICO result. FORCE provides cleaner dispersion patterns with less noise, especially near tissue boundaries. AMICO shows low dispersion in fluid-filled spaces where it is expected to be higher.

The comparison was extended to multi-compartment modeling by evaluating FORCE against AMICO for NODDI parameter estimation (Figure 5). FORCE yields ODI maps that are more anatomically consistent and less noisy than AMICO, supported by a stronger correlation with inverted T1w used to match ODI contrast (FORCE: r=0.9296; AMICO: r=0.8231). In regions with substantial fiber crossing, AMICO reports higher ODI whereas FORCE reports lower ODI, likely because AMICO assumes a single fiber population per voxel while FORCE models multiple fascicles explicitly, reducing confounding effects by fiber count. For NDI, FORCE produces sharper anatomical detail and corrects artifacts in the AMICO result, which erroneously identifies CSF-filled ventricles as having high neurite density; the NDI–T1w correlation is also higher for FORCE (FORCE: r=0.8957; AMICO: r=0.8791). Finally, the free-water (FW) map from FORCE is cleaner, delineating fluid-filled spaces such as the ventricles and the subarachnoid space more precisely. Notably, the AMICO FW map shows higher values than FORCE. This is because AMICO’s NODDI implementation uses a fixed intrinsic parallel diffusivity $\left(1.7\times 10^{-3}{\text{mm}}^{2}/\text{s}\right)$. When actual tissue diffusivity is lower, the model compensates by attributing the extra diffusivity to a higher FW content, leading to an overestimation of FW. This comparison therefore highlights the inherent advantage of our flexible approach in avoiding such assumption driven errors. Also, low ambiguity in coherent white matter regions such as the corpus callosum indicates a unique, well-defined match, consistent with high FA values (Figure C30). Whereas elevated uncertainty is confined to gray and white matter interfaces, reflecting natural partial-volume mixing (Figure C29). These observations confirm that evaluating a local neighborhood of K=50 candidates is sufficient to capture realistic degeneracies while maintaining anatomical specificity and robust uniqueness of matching.

**Fig. 6:**
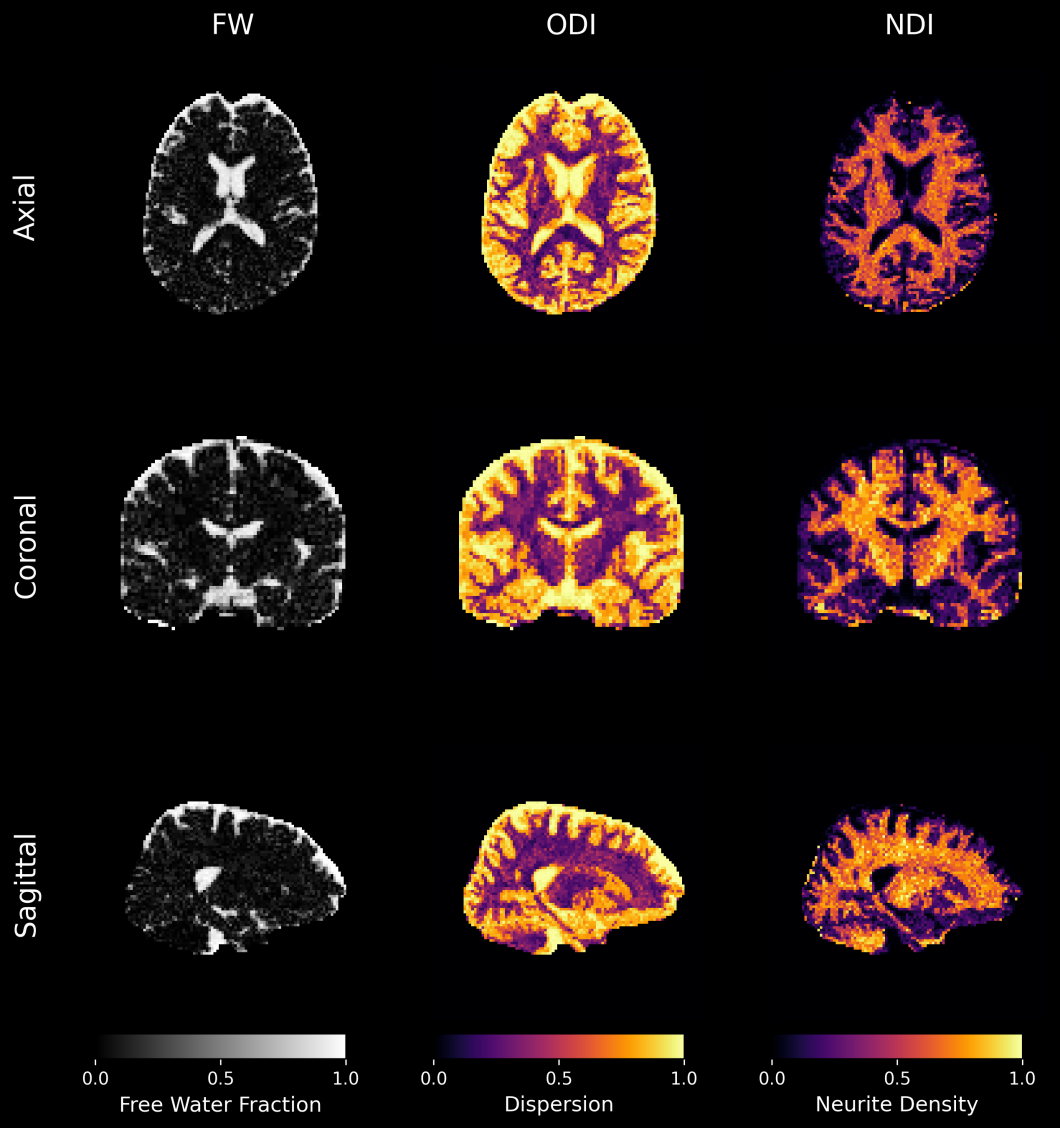
NODDI parameter maps generated by FORCE from the single-shell Stanford HARDI dataset. The results demonstrate the method’s capability to estimate complex microstructural indices from acquisitions that lack multiple b-values and have lower voxel resolution which in this case is roughly 4 times bigger than a HCP 3T dataset voxel.

**Fig. 7:**
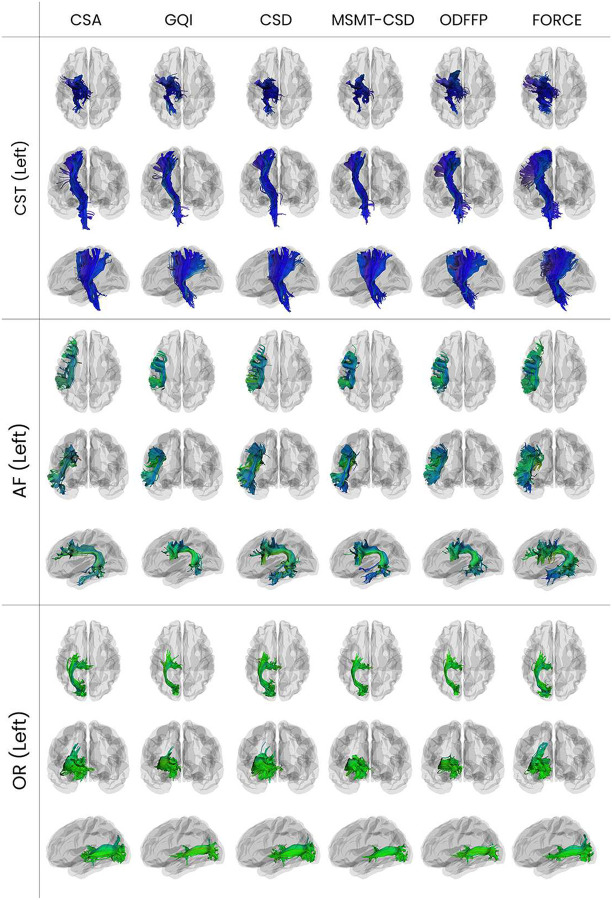
Renderings of the left corticospinal tract (top), left arcuate fasciculus (middle), left optic radiation (bottom) using six different reconstruction algorithms: CSA, GQI, CSD, MSMT-CSD, ODFFP, and FORCE and extracted using RecoBundles with default parameters. Tracts are displayed from Axial (top), Coronal (middle), and Sagittal (bottom) views within each panel.

FORCE can yield robust estimates even from single-shell data such as the Stanford HARDI dataset, which are not ideal for models like NODDI that require multi-shell acquisitions to estimate microstructural parameters accurately. As shown in Figure 6, FORCE generated plausible microstructural maps for FW fraction, dispersion (ODI), and ND. This highlights the method’s versatility and its capacity to regularize the model fitting to provide stable estimates from more limited, single-shell acquisitions. The anatomical plausibility of FORCE’s reconstructions is further highlighted by direct visual comparison with MSMT-CSD, a state-of-the-art inverse modeling technique. As shown in Figure C5, in regions with simple, coherent architecture like the corpus callosum, FORCE generates clean, single-fiber orientations that are qualitatively indistinguishable from those produced by MSMT-CSD. Critically, in more challenging areas with complex crossing fibers, FORCE demonstrates a comparable ability to resolve the multiple underlying populations. This consistency provides strong evidence that operating directly in the signal space with a biologically plausible simulation of signals allows FORCE to achieve an accuracy competitive to established deconvolution methods, reinforcing its utility as a robust alternative for mapping complex white matter pathways. The anatomical plausibility of the FORCE reconstructions is evident from the extracted bundles shown in Figure 7.

### 3.4 Ex vivo Diffusion Data

FORCE is evaluated on two ex vivo datasets to assess its robustness under altered microstructural environments and reduced diffusivity:

- **Mouse brain** [72]: Single-shell acquisition at $b=4000\;\text{s}/{\text{mm}}^{2}$ with 46 diffusion-weighted images (DWIs) and 5 $b_{0}$ volumes; voxel size $0.055\times 0.055\times 0.055\;{\text{mm}}^{3}$ (isotropic).
- **Human brain (ex vivo)**: Multi-shell acquisition with $b_{0}$ and four nonzero b-value shells $\left(b\approx 1000,2000,3000,4000\;\text{s}/{\text{mm}}^{2}\right)$; voxel size $2\times 2\times 2\;{\text{mm}}^{3}$ (isotropic).

Using a $b_{0}$ threshold of $50\;\text{s}/{\text{mm}}^{2}$ (volumes with $b<50\;\text{s}/{\text{mm}}^{2}$ were treated as $b_{0}$), 34 $b_{0}$ images were identified. The diffusion-weighted images were grouped into four approximate shells $\left(b\approx 1000,2000,3000,4000\;\text{s}/{\text{mm}}^{2}\right)$. FORCE reproduced diffusion tensor and kurtosis contrasts, showing high anisotropy with low mean diffusivity along major white-matter pathways and elevated kurtosis in regions of complex fiber architecture, consistent with expected behavior.

For NODDI maps, AMICO and FORCE produced broadly similar results, with differences concentrated near tissue boundaries. In the NDI map, both methods show clear GM/WM contrast and visible structure within white matter; FORCE is slightly sharper, and the residuals are small (Figure C8, top row). For the ODI map, AMICO exhibits fluctuations at CSF/WM interfaces that are reduced by FORCE, yielding smoother and more anatomically coherent patterns across boundaries (Figure C8, middle row). FW fraction maps are highly consistent between methods, with high values confined to fluid-filled spaces and only minor residuals (Figure C8, bottom row).

FORCE was additionally applied to the high-resolution ex vivo mouse brain dataset to assess its performance under single-shell conditions. Despite the limited diffusion weighting, FORCE produced high-quality DTI maps with strong contrast between anisotropic and isotropic regions, demonstrating reliable reconstruction of diffusion tensor features under reduced diffusivity (Figure C9).

Beyond DTI, FORCE generated stable and anatomically coherent NODDI maps from the same single-shell data (Figure C10). The NDI and ODI maps exhibited smooth spatial variation with consistent gray–white contrast, while the FW fraction remained low throughout the tissue, consistent with minimal free water and more restricted diffusion in ex vivo specimens. These results highlight FORCE’s capacity to extract reliable and biologically meaningful diffusion and microstructural metrics from high-resolution, limited ex vivo acquisitions.

### 3.5 Glioma and Parkinson’s disease (PPMI)

To evaluate clinical applicability, diffusion MRI data from patients with gliomas were analyzed [73]. The dataset comprises pre-surgical diffusion-weighted scans at clinical resolution ($b=1000\;\text{s}/{\text{mm}}^{2}$, 64 directions; voxel size $2\times 2\times 2\;{\text{mm}}^{3}$). As shown in Figure C11, FORCE closely reproduces standard DTI metrics, yielding FA, MD, and RD maps that are nearly identical to conventional tensor fitting, with minimal residuals even in peritumoral regions. Figure C12 presents FORCE-derived FW, ODI, and NDI maps. The maps show increased FW and reduced ND in peritumoral areas, consistent with extracellular fluid accumulation and tissue disorganization, and elevated dispersion in regions of disrupted fiber architecture.

In addition, a subset of diffusion MRI scans from the Parkinson’s Progression Markers Initiative (PPMI) [74] was analyzed (single-shell $=1000\;\text{s}/{\text{mm}}^{2}$, 64 directions; voxel size $1.98\times 1.98\times 2\;{\text{mm}}^{3}$). FORCE outputs were processed with the BUAN tractometry pipeline to derive bundle-level measures. Figures C13 and C14 show segmental significance profiles for FA, MD, and RD across four major bundles in the left and right hemispheres, respectively, with spatial patterns consistent with prior BUAN findings [75].

## 4 Discussion

This work introduced FORCE, a forward modeling framework for fiber orientation and microstructure estimation that directly operates in signal space. The results demonstrate that FORCE provides performance comparable to or exceeding that of traditional and state-of-the-art methods under diverse acquisition conditions.

Simulation experiments demonstrate that FORCE is capable of resolving shallow fiber crossings in the 10° – 40° range, a regime where many ODF-based methods begin to lose reliability (Figure 3). ODFFP is effective in identifying low-angle crossings but overestimates number of fibers with dispersion. In contrast, FORCE directly simulates the diffusion signal using a forward model that accounts for dispersion, allowing it to maintain robustness in challenging configurations while being a more computationally efficient alternative (Figure 2). The close agreement between simulated and measured signal profiles, as reflected in DTI metrics (Figures C6, C7) that depend solely on the signal, indicates that extreme oversampling of the simulation space yields diminishing returns

Importantly, FORCE not only identifies fiber orientations but simultaneously estimates biologically meaningful microstructural parameters such as DTI, DKI, NODDI maps and provides soft tissue segmentation. Traditional models like DTI or CSD require separate pipelines or modeling assumptions for such features, while FORCE yields them directly from the best-matching configuration without additional fitting (Figures 4, 5, C1).

These results support the practical utility of FORCE in tractography-based pipelines and connectivity analyses (Figure 7). FORCE produced consistent reconstructions across acquisition paradigms, species, clinical settings, despite differences in voxel size, angular resolution of acquisitions, and b-values. The reconstructed orientation maps and derived microstructural metrics aligned well with known anatomical structures across brain regions (Figures 6, C3, C4, C8, C9, C10, C11, C12). In addition, FORCE integrates with standard tractometry pipelines. BUAN analyses on FORCE-derived bundles and microstructure maps produced anatomically plausible segmental significance profiles, supporting group-wise inference from the same single forward fit (Figures C13, C14). FORCE also quantifies signal-space degeneracy with two voxel-wise maps: uncertainty measures the spread of plausible matches and ambiguity measures the width of the similarity peak; empirically, ambiguity is low in coherent white matter such as the corpus callosum, while uncertainty is elevated at GM/WM interfaces consistent with partial volume (Figures C29, C30).

FORCE processes each voxel independently so the entire pipeline is inherently parallelizable, allowing efficient processing of large datasets across multiple CPU/GPU threads or compute nodes. A detailed runtime comparison (Figure C16) demonstrates that FORCE achieves faster performance than current state-of-the-art methods such as MSMT-CSD. Furthermore, FORCE offers higher computational efficiency compared to executing multiple existing models to derive equivalent microstructural parameters. Additional acceleration can be achieved through an approximate nearest-neighbor search using locality-sensitive hashing (LSH), which introduces a small approximation error in exchange for faster retrieval, referred to as FORCE-ACC (Appendix B)

Despite its versatility, FORCE has certain limitations. The reliance on large number of precomputed simulations imposes memory and computational demands, particularly as the dimensionality of signal space increases. Also, because the simulations are generated through random sampling of the parameters, the parameter space remains inherently undersampled, capturing a diverse but non-exhaustive range of configurations. While cosine similarity offers a robust and interpretable matching criterion, future improvements in scalability may arise from incorporating approximate search strategies or compressed representations of simulated signals. In addition, discrete angular sampling imposes an upper bound on achievable orientation resolution, which could be mitigated through adaptive simulation refinement or interpolation-based extensions. The matching is also sensitive to noise at lower SNR since the signals along fiber directions have lower signal magnitude.

Future developments will aim to extend FORCE beyond the brain to characterize diffusion processes in other organs and tissues. Future work will also explore the extension of FORCE in double diffusion encoding acquisitions. Enhancements in computational efficiency, spatial integration, and model adaptability are expected to further expand its applicability.

## 5 Conclusion

FORCE is a forward modeling framework for fiber orientation and microstructure estimation that operates directly in signal space via simulation-driven cosine matching. Unlike conventional inverse reconstructions, FORCE eliminates model-fitting and projection steps by matching biophysically grounded simulated signals to the measurements to infer orientation and tissue properties. Across synthetic, phantom, in vivo, and ex vivo datasets spanning multiple species, single and multi-shell acquisitions, and varied spatial resolutions, FORCE consistently recovers complex fiber configurations and remains robust to noise and acquisition variability. Despite this breadth, it achieves high computational efficiency via parallelized search. A key advantage of FORCE is that it unifies orientation peaks, tissue segmentation, and DTI/DKI/NODDI maps from a single simulation–match step, removing the need to combine multiple model pipelines. These findings show that a single biophysical model, coupled with scale-invariant matching, can span multiple levels of diffusion representation without sacrificing interpretability. Together, these results position FORCE as a biologically grounded, computationally efficient alternative to inverse-model reconstructions for routine dMRI.

## Supplementary Material

1

## Figures and Tables

**Table 1:** Parameter sampling ranges and distributions used for signal simulations.

Parameter	Distribution / Values
WM/GM/FW fractions	Dirichlet(2, 1, 1)
Number of fibers N	{0, 1, 2, 3}
Orientations	Uniform on $S^{2}$ (724-vertex grid)
ODI (WM)	Equispaced on [0.01, 0.30](n=10)
ODI (GM/FW)	1 (fully isotropic)
Parallel diffusivity (in vivo) $D_{\|}^{\text{in}}=D_{\|}^{\text{ex}}$	$\text{Uniform}(2.0, 3.0)\times 10^{-3}\;{\text{mm}}^{2}/\text{s}$
Perpendicular extra-axonal diffusivity (in vivo) $D_{⊥}^{\text{ex}}$	$\text{Uniform}(0.1, 1.5)\times 10^{-3}\;{\text{mm}}^{2}/\text{s}$
Parallel diffusivity (ex vivo) $D_{\|}^{\text{in}}=D_{\|}^{\text{ex}}$	$\text{Uniform}(0.6, 1.0)\times 10^{-3}\;{\text{mm}}^{2}/\text{s}$
Perpendicular (ex vivo) $D_{⊥}^{\text{ex}}$	$\text{Uniform}(0.01, 0.35)\times 10^{-3}\;{\text{mm}}^{2}/\text{s}$
Intra-axonal model	Stick model ($D_{⊥}^{\text{in}}=0$)
Intra-axonal fraction	Uniform(0.6, 0.9)
Fiber weights (2 fibers)	Uniform(0.2, 0.8)
Fiber weights (3 fibers)	Dirichlet(1, 1, 1)
Gray matter diffusivity $D_{\text{GM}}$	$1\times 10^{-3}\;{\text{mm}}^{2}/\text{s}$
Free-water diffusivity $D_{\text{FW}}$	$3\times 10^{-3}\;{\text{mm}}^{2}/\text{s}$

## Data Availability

Public datasets were used in this study. These can be accessed at the following URLs:
HCP: https://db.humanconnectome.org/ Stanford HARDI: https://purl.stanford.edu/ng782rw8378
Additional data used in the study can be provided upon request.

## Materials availability

All materials used in this study are commercially available or described in the Methods section.

## Code availability

The code will be made publicly available in the DIPY (Diffusion Imaging in Python) package (https://www.dipy.org) upon publication.
