# Supplementary material for: FORCE: FORward modeling for Complex microstructure Estimation
Source: Res Sq. 2025 Nov 20:rs.3.rs-8151109. Preprint. [Version 1] doi: 10.21203/rs.3.rs-8151109/v1 (PMC12668119; doi:10.21203/rs.3.rs-8151109/v1)
Supplement: 1 [file NIHPPRS8151109V1-supplement-1.pdf]

Appendix A Analytical formulation of DKI parameters

In the presented approach, fitting the diffusional kurtosis imaging (DKI) model directly to the synthetic signals generated by the forward model can be avoided. Instead, the analytical solution of the diffusion and kurtosis tensors can be used to estimate directly while simulation, following the same strategy implemented in DIPY [18]. As all the required parameters are sampled, an analytical solution to the DKI parameters can be found by avoiding the expensive fitting procedure.

For a system of N Gaussian diffusion compartments, each defined by a water fraction f_m and diffusion tensor $D_{ij}^{(m)}$, the composite diffusion tensor is simply the weighted sum of the compartmental tensors:

$$D_{ij} = \sum_{m=1}^{N} f_m D_{ij}^{(m)}. \tag{A1}$$

The corresponding kurtosis tensor can then be computed analytically as:

$$W_{ijkl} = \frac{1}{MD^2} \left(\sum_{m=1}^{N} f_m \left[D_{ij}^{(m)} D_{kl}^{(m)} + D_{ik}^{(m)} D_{jl}^{(m)} + D_{il}^{(m)} D_{jk}^{(m)} \right] - D_{ij}D_{kl} - D_{ik}D_{jl} - D_{il}D_{jk} \right), \tag{A2}$$

where $MD = \frac{1}{3}\mathrm{Tr}(D)$ denotes the mean diffusivity. Equations (A1–A2) provide a closed-form mapping between diffusion and kurtosis terms, so the DKI parameters are well defined by the model and can be computed analytically in a multicompartment simulation framework.

Appendix B Approximate Nearest Neighbor Search

Locality-Sensitive Hashing (LSH)

Approximate nearest neighbor (ANN) methods accelerate search in high-dimensional spaces by exploiting the principle of locality-sensitive hashing. For cosine similarity, one constructs hash functions based on random hyperplanes. Each bit of the hash code is the sign of a dot product with a random vector. If $x, y \in \mathbb{R}^d$ are unit vectors separated by angle $\theta(x, y)$, then

$$\Pr[h(x) = h(y)] = 1 - \tfrac{\theta(x,y)}{\pi}.$$

Thus, similar vectors are more likely to collide in hash space, while dissimilar vectors are less likely. Increasing the number of hash bits b sharpens discrimination, concentrating the Hamming distance around the true angular separation.

Hadamard Projection

Prior to hashing, one may apply a randomized Hadamard transform: multiply each coordinate of the vector by a random ± 1 sign and apply a fast Hadamard transform (FHT). This acts as a nearly orthogonal rotation with two benefits: (i) it spreads energy uniformly across coordinates, improving the quality of subsequent LSH; and (ii) it is computationally efficient, requiring $O(d \log d)$ operations. The result is a projection that preserves angular similarity while allowing very fast hashing.

Voronoi Cells and Exact Guarantees

Let $D = \{s_1, \ldots, s_N\}$ be the set of simulated signals on the unit sphere. Each s_i defines a Voronoi cell

$$V_i = \{x \in \mathbb{S}^{d-1} : \|x - s_i\| \leq \|x - s_j\| \;\; \forall j\}.$$

If D is an ε-net of the signal manifold $\mathcal{M}$, then every voxel signal $x \in \mathcal{M}$ has a neighbor $s \in D$ with $\|x - s\| \leq \varepsilon$. Since

$$\|x - s\|^2 = 2(1 - \cos(x, s)),$$

this implies a cosine similarity lower bound

$$\cos(x, s) \geq 1 - \tfrac{\varepsilon^2}{2}.$$

The covering number $N(\varepsilon, \mathcal{M})$ gives the smallest N required for such an ε-net. For a manifold of intrinsic dimension m,

$$N(\varepsilon, \mathcal{M}) \lesssim C\varepsilon^{-m} \quad \Longrightarrow \quad \varepsilon \lesssim \left(\tfrac{C}{N}\right)^{1/m}.$$

Thus, increasing Voronoi cells shrinks the worst-case approximation error at rate $N^{-1/m}$.

Probability of retrieval error in ANN

ANN modifies this guarantee by introducing a probability of missing the true nearest neighbor. For LSH with b bits per table and L independent tables, the probability that two vectors x, y with similarity $s = \cos\theta$ collide in at least one table is

$$p_{\mathrm{hit}} = 1 - (1 - s^b)^L.$$

The failure probability is therefore

$$p_{\mathrm{miss}} = (1 - s^b)^L.$$

As b increases, collisions concentrate more sharply around true neighbors; as L increases, the chance of at least one successful collision grows. By retrieving a candidate set from these collisions and re-ranking with exact cosine similarity, the only source of error is p_{miss}, which can be made arbitrarily small.

Connecting Voronoi Cells to ANN Guarantees

When the dictionary is sufficiently dense (small ε), brute-force matching guarantees a neighbor within ε, hence cosine similarity at least $1 - \varepsilon^2/2$. ANN inherits this guarantee up to the failure probability p_{miss}. As the number of Voronoi cells grows ($N \uparrow$), ε shrinks as $N^{-1/m}$, so the match becomes more accurate. At the same time, with properly tuned LSH parameters (b, L), the probability of missing the correct Voronoi cell becomes negligible. Thus, increasing the number of Voronoi cells improves accuracy both geometrically (smaller ε) and probabilistically (lower p_{miss}), ensuring that ANN results are practically indistinguishable from exact nearest neighbor search.

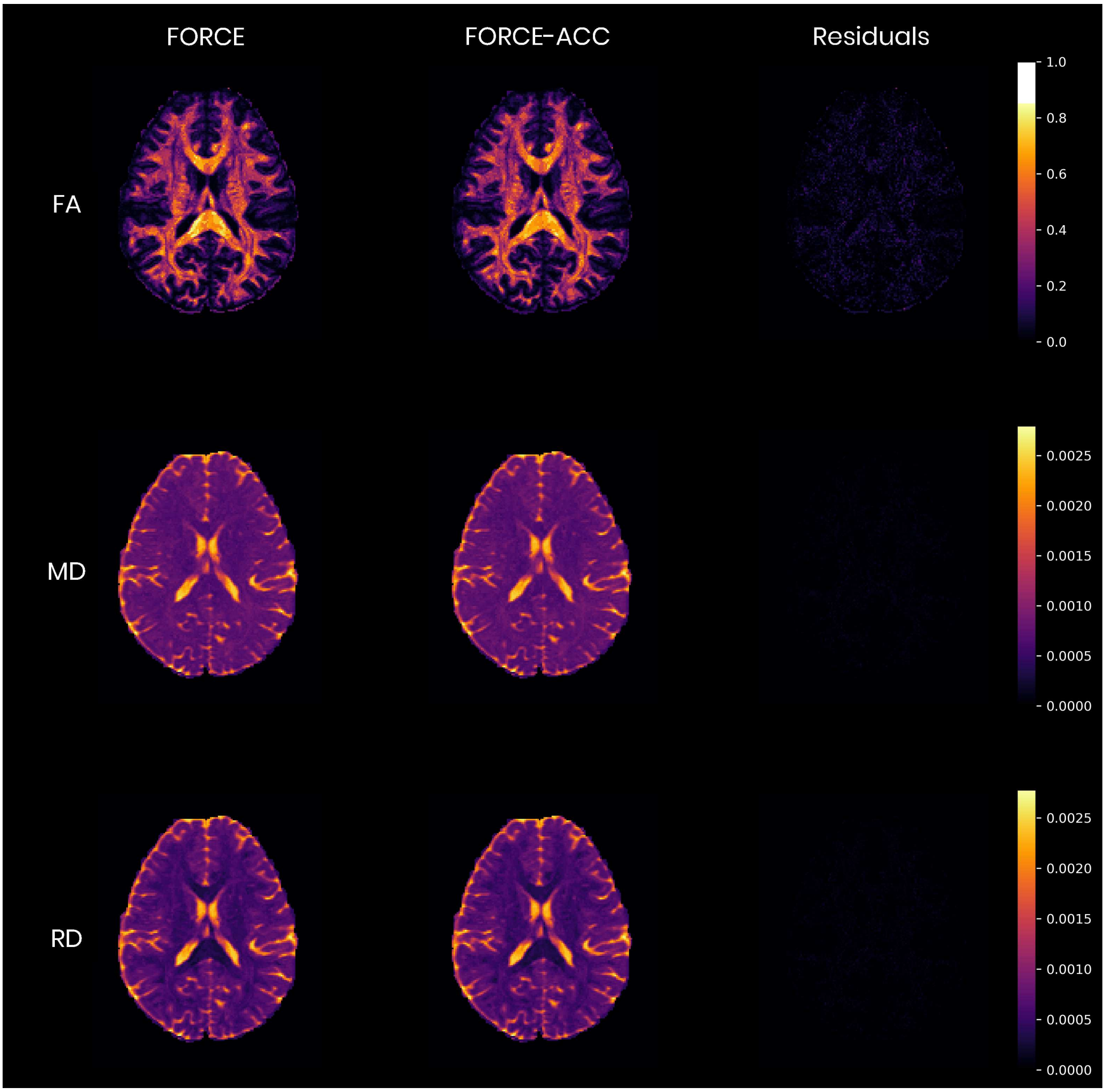

Fig. B1 Comparison of DTI microstructure maps from FORCE with exact match and FORCE accelerated with LSH with 1000 Voronoi cells. The difference is a small tradeoff for the $\sim 50\%$ time reduction in matching.

Appendix C Additional figures

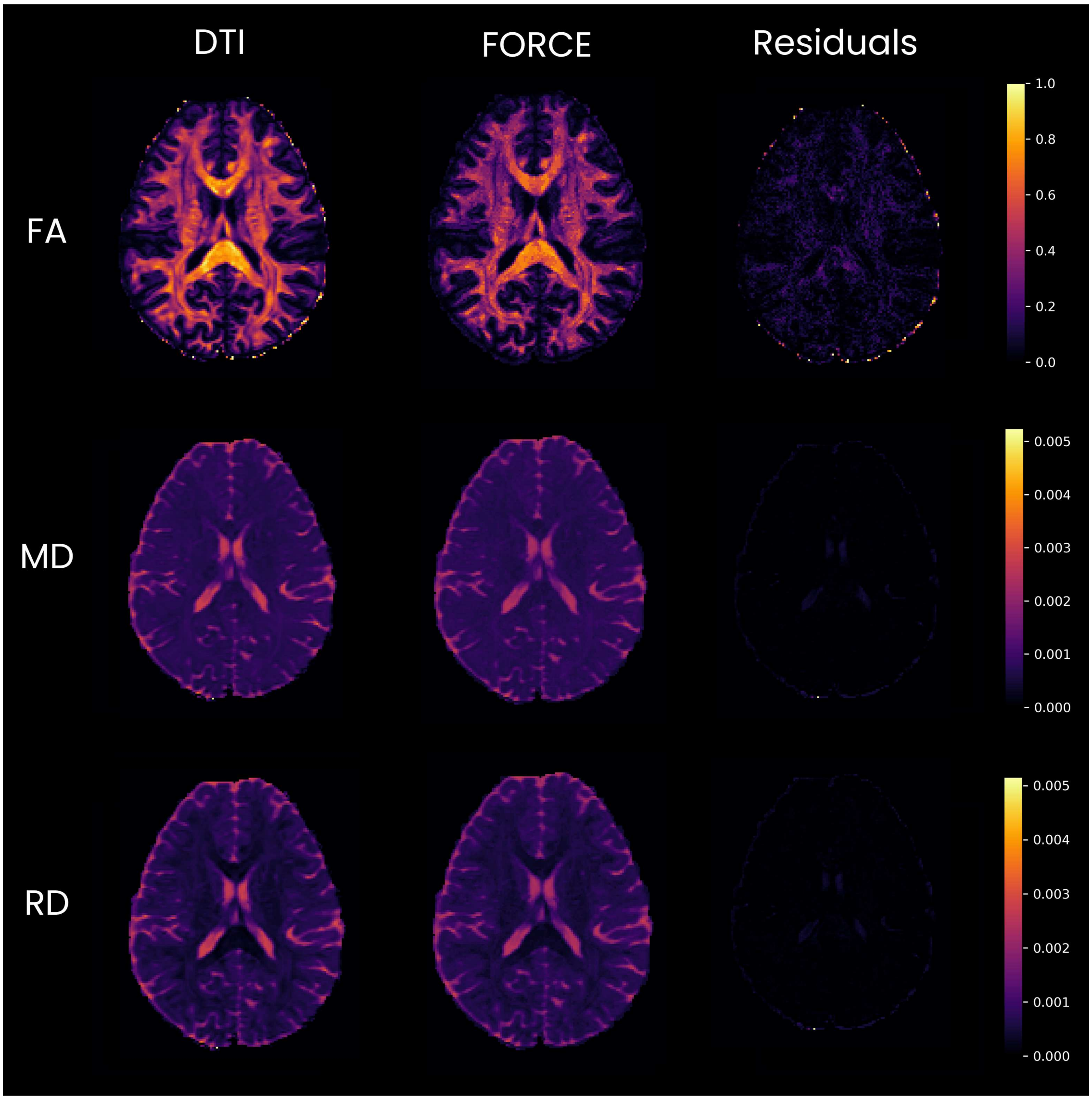

Fig. C1 Comparison of DTI parametric maps from conventional fitting (left) and FORCE (middle) on the HCP 3T dataset. The absolute difference maps (right) highlight the difference. The low residuals across all metrics highlight that FORCE closely preserves the diffusion signal characteristics while providing consistent agreement with standard DTI measures.

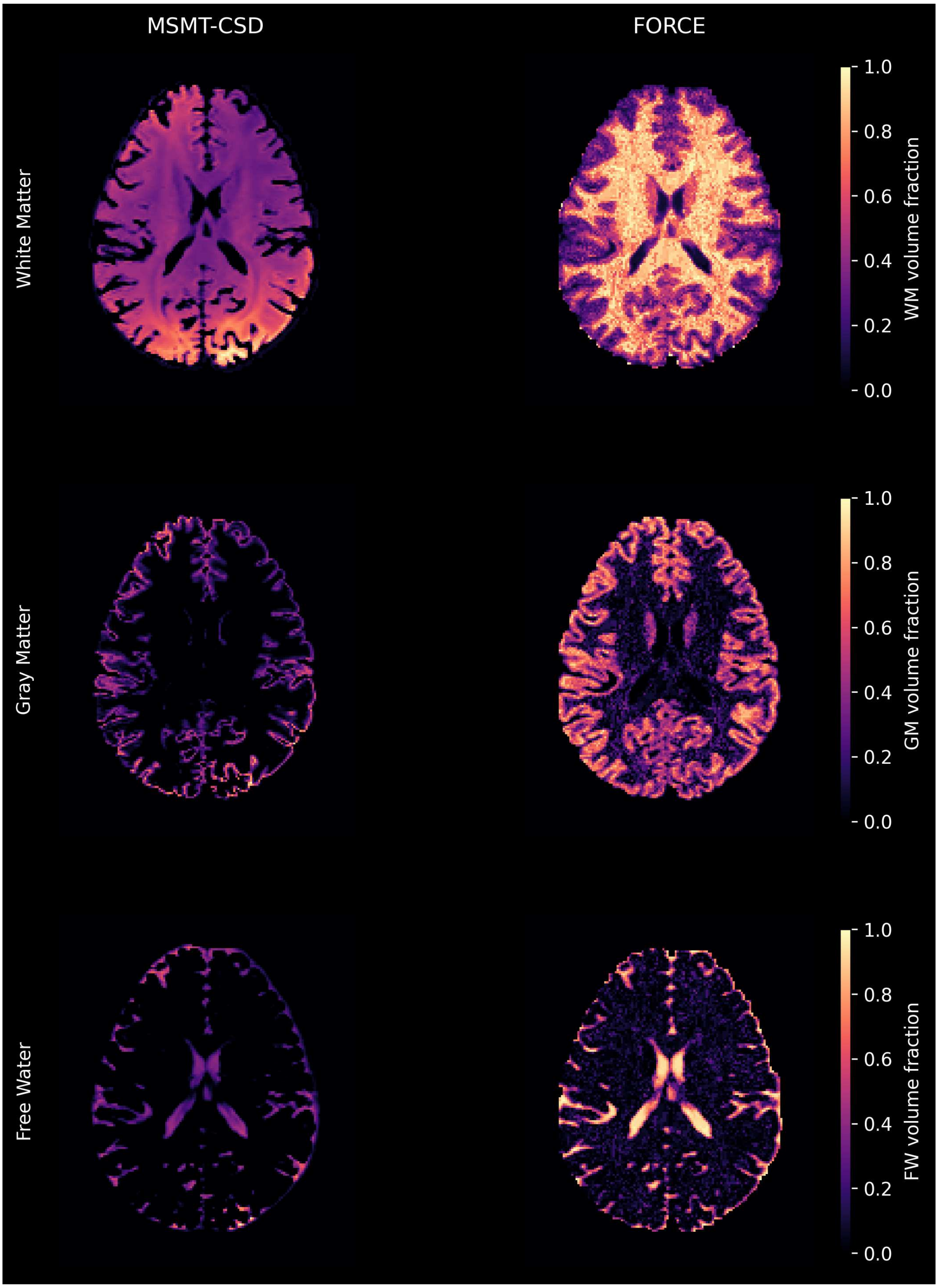

Fig. C2 Anatomical comparison of tissue volume fractions estimated by MSMT-CSD and FORCE. FORCE (right column) exhibits higher contrast in deep white matter structures and recovers a more prominent cortical gray matter ribbon compared to MSMT-CSD (left column). Additionally, the FW map from FORCE reveals more extensive cerebrospinal fluid distribution within the cortical sulci. (MSMT-CSD fractions normalized between 0 and 1).

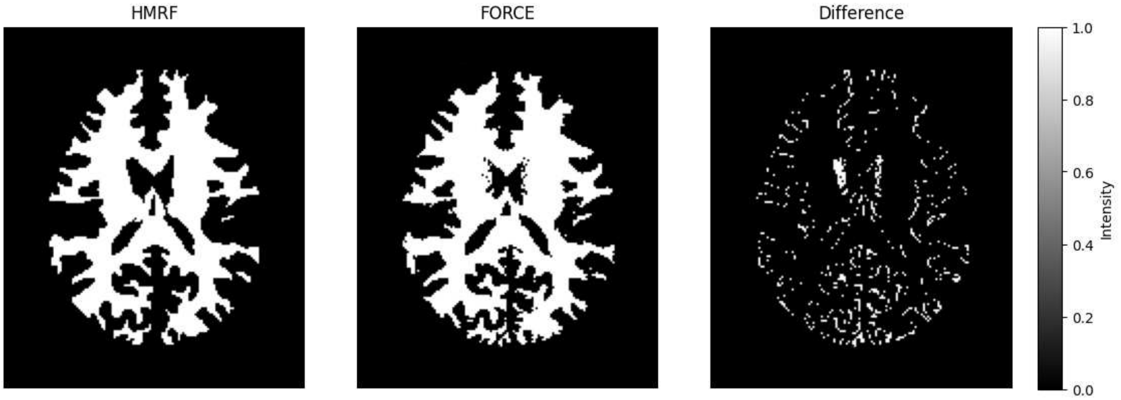

Fig. C3 Comparison of white matter (WM) segmentations derived from the Hidden Markov Random Field (HMRF) method (left) on T1w image and the FORCE algorithm binarized with a threshold of 0.5 (middle). The right panel displays the absolute difference map, highlighting areas of disagreement.

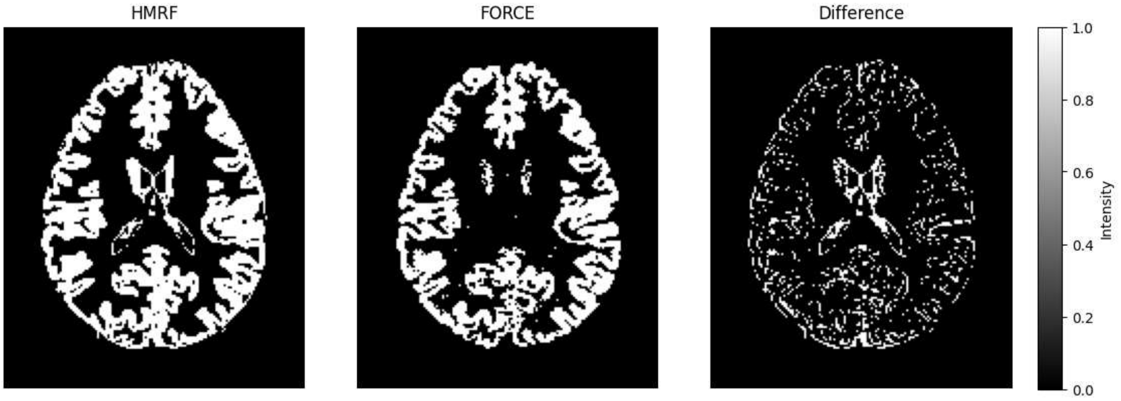

Fig. C4 Comparison of gray matter (GM) segmentations derived from the Hidden Markov Random Field (HMRF) method (left) on T1w image and the FORCE algorithm binarized with a threshold of 0.5 (middle). The right panel displays the absolute difference map, highlighting areas of disagreement. The difference map (right) highlights significant disagreement at the cortical-CSF boundary. At this interface, FORCE provides a sharper separation, while the HMRF result shows signs of over-segmentation due to partial volume effects.

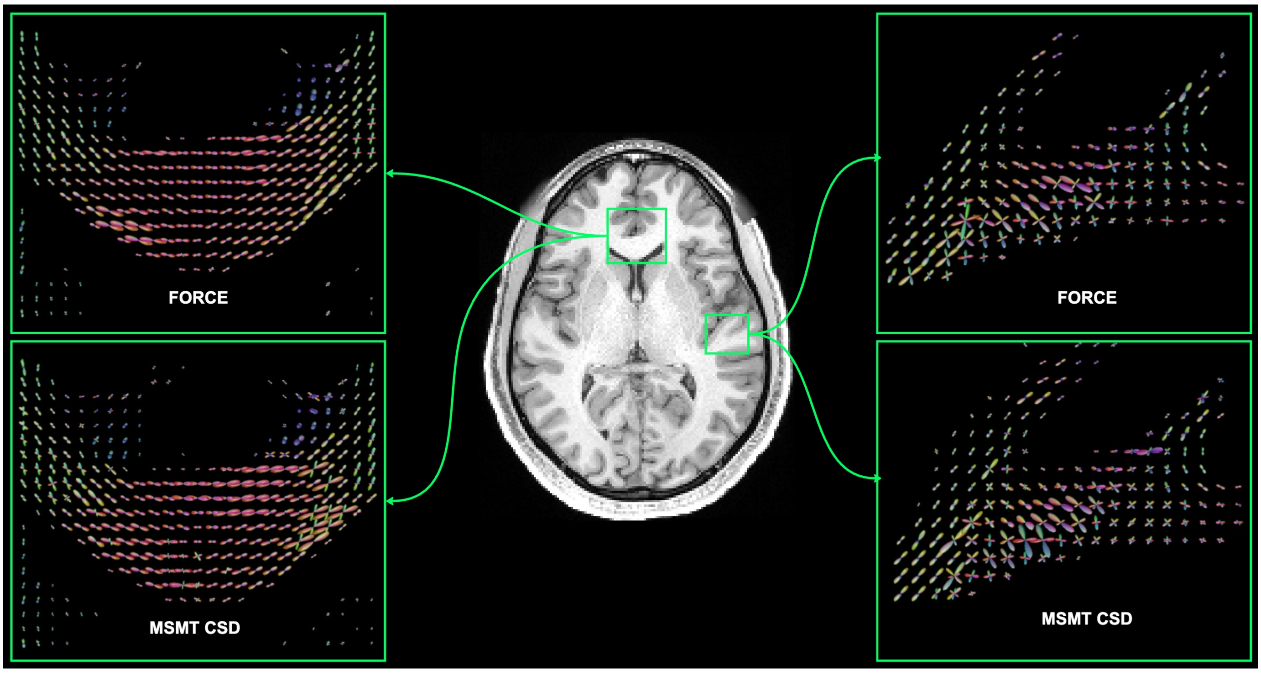

Fig. C5 Comparison of Orientation Distribution Functions (ODFs) reconstructed with FORCE (top) and Multi-Shell, Multi-Tissue Constrained Spherical Deconvolution (MSMT-CSD) (bottom) on HCP 3T dataset with an isotropic voxel size of $1.25 \times 1.25 \times 1.25$ mm^3. The central T1w anatomical image shows the location of two selected white matter regions. The left insets display a region with highly coherent fibers (corpus callosum), while the right insets show a region with complex crossing fibers and tissue boundaries. Consistent with the tracking results, FORCE dODFs (diffusion ODFs) show fewer crossings in middle of corpus callosum (left) and more at the lateral intersection areas (right). Although dODFs are typically broader, FORCE yields sharply peaked dODFs, which are visually comparable to fiber-ODFs (fODFs) without any deconvolution process used.

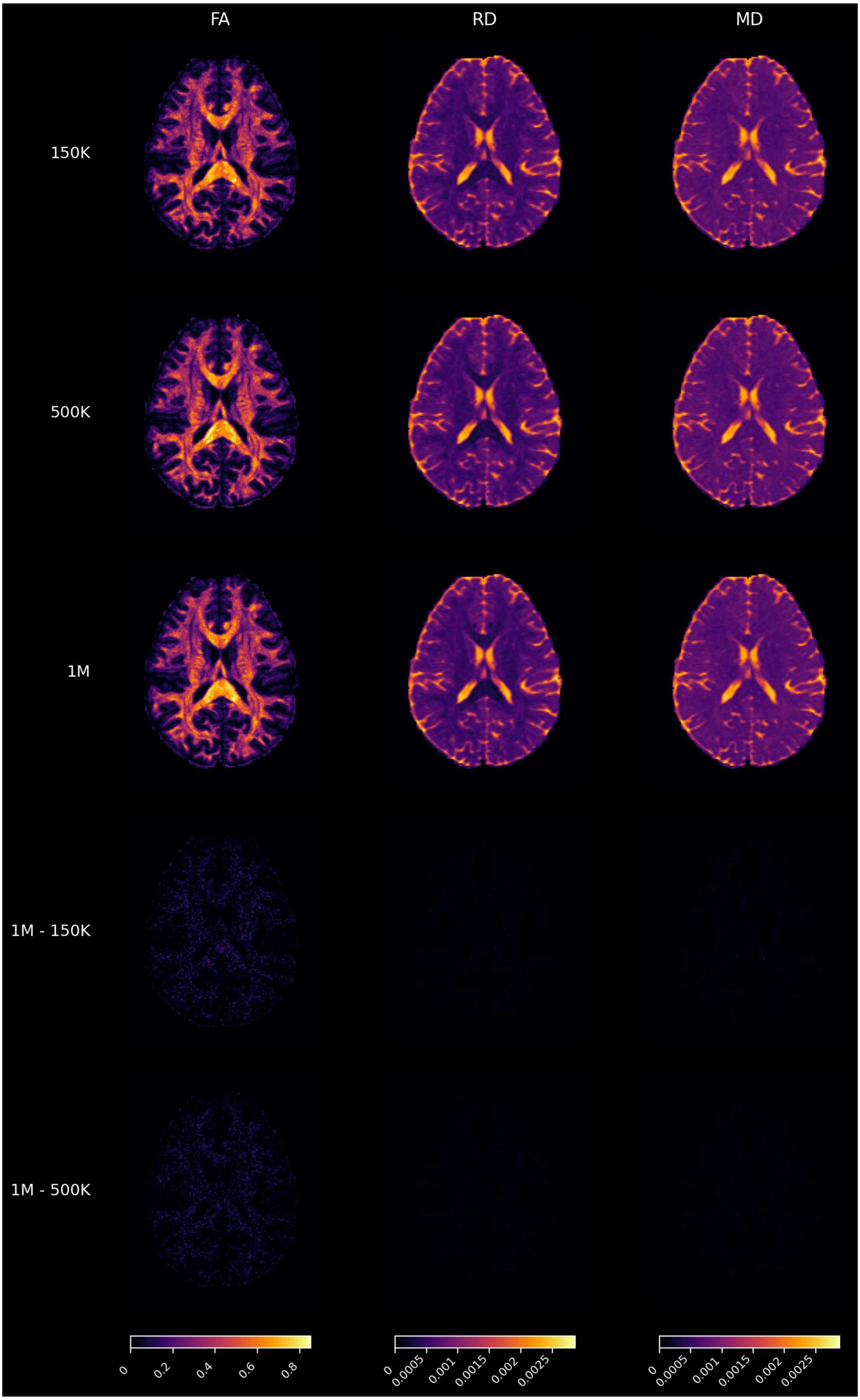

Fig. C6 The maps illustrate FA, RD, and MD as the simulation size increases. The top three rows show the estimated maps for 150K, 500K, and 1M simulations, respectively. The bottom two rows display the absolute difference maps between the 1M reference map and the maps from the smaller dictionaries. The progressive reduction in error, particularly the minimal difference shown in the "1M - 500K" row is the reason of choosing 500K as the default option.

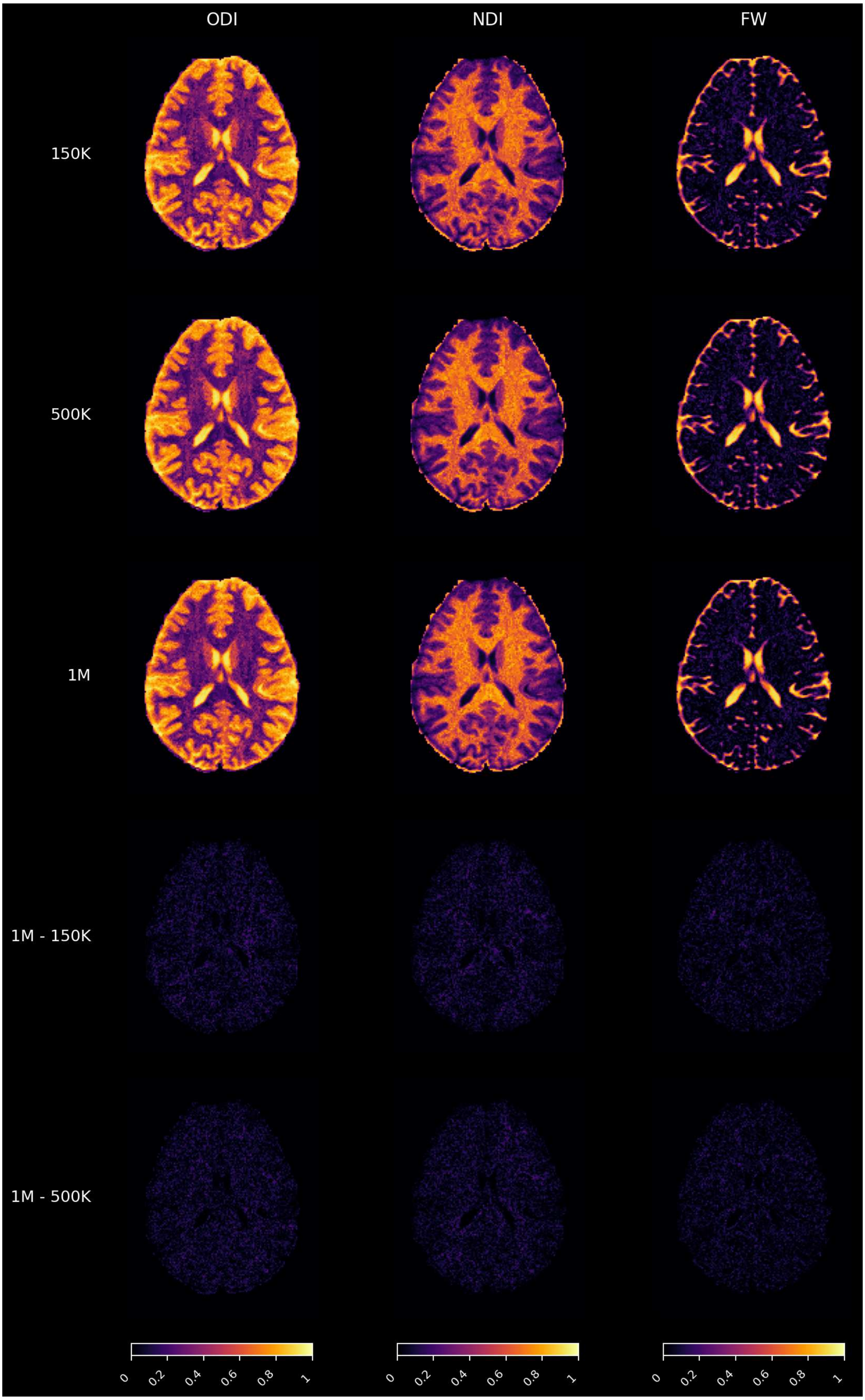

Fig. C7 The maps illustrate ODI, NDI, and FW as the simulation size increases. The top three rows show the estimated maps for 150K, 500K, and 1M simulations, respectively. The bottom two rows display the absolute difference maps between the 1M reference map and the maps from the smaller dictionaries.

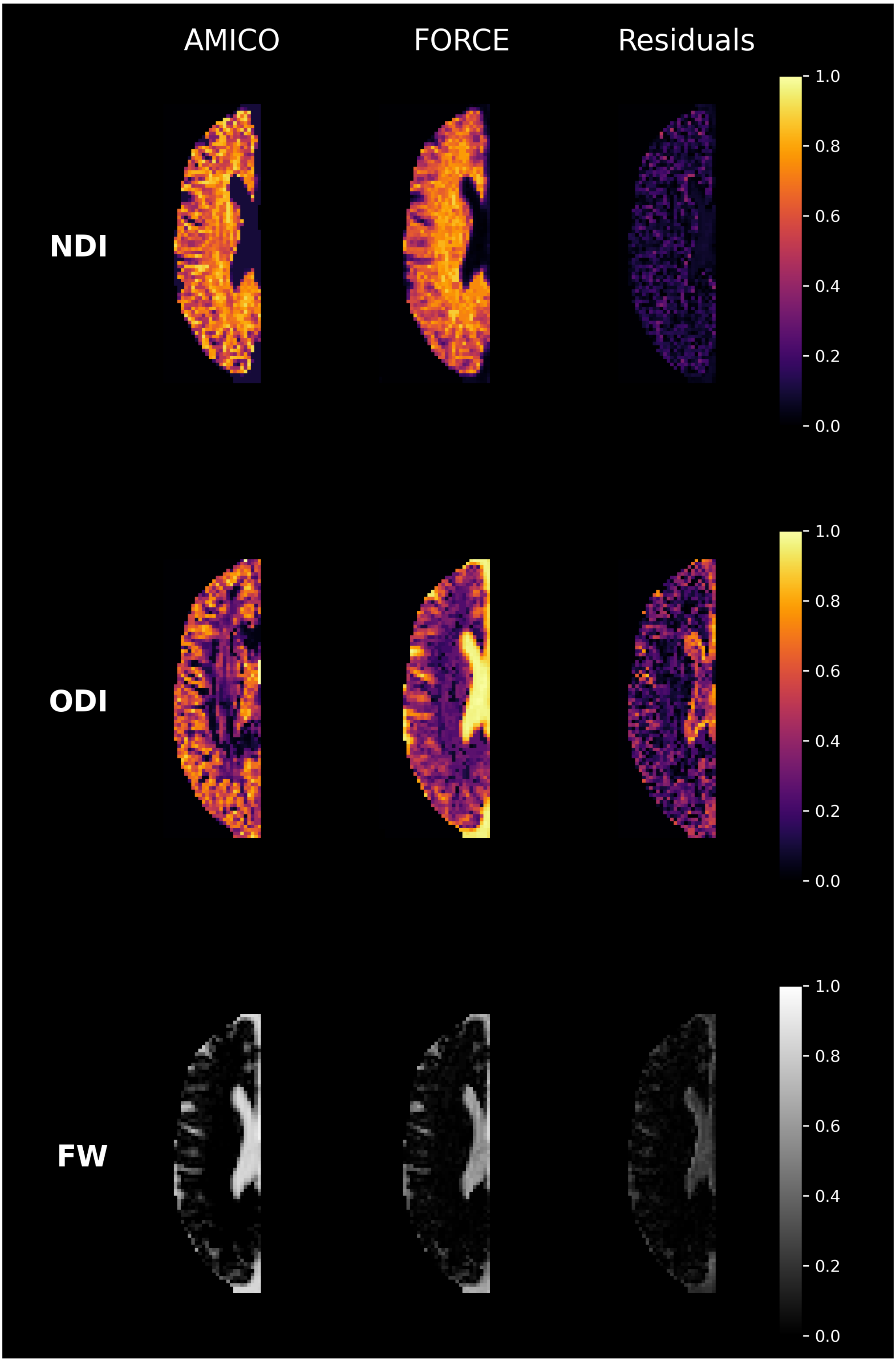

Fig. C8 Ex vivo post-mortem dMRI of the left hemisphere preserved in formalin and imaged inside a plastic jar. Despite the restricted diffusion regime in this acquisition, FORCE produces smoother and more biologically plausible parameter maps compared to AMICO's ex vivo implementation with range appropriate parameters. Shown are NDI (top row), ODI (middle row), and FW (bottom row) for AMICO (left), FORCE (middle), and the corresponding residuals (right). FORCE recovers meaningful tissue contrast and smoother transitions across cortical and subcortical regions, while AMICO maps appear noisier with elevated residuals.

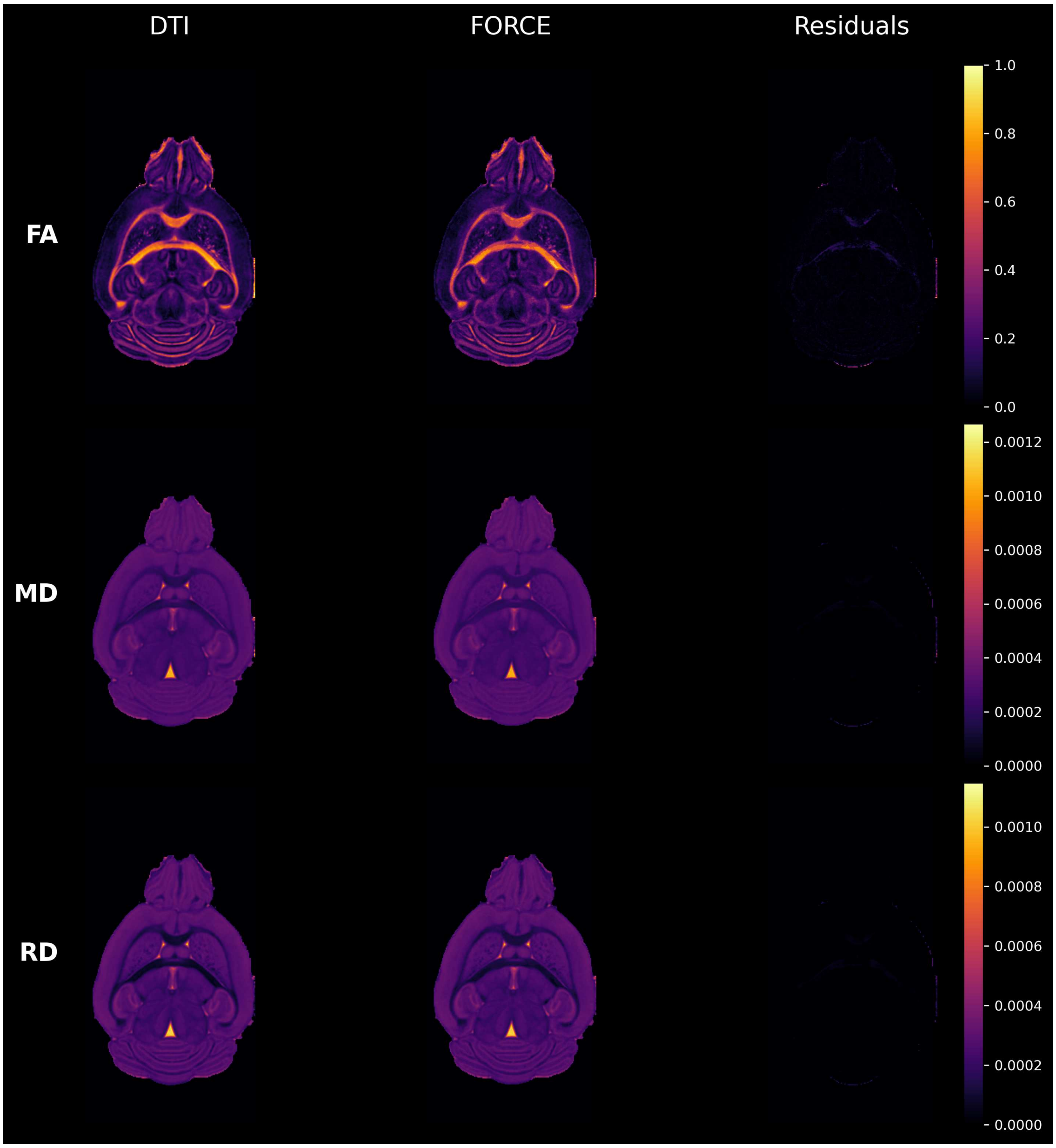

Fig. C9 Comparison of metrics between standard DTI and FORCE in mice. Axial slices show FA, MD, and RD maps (rows). For each metric, the left column shows DTI, the middle shows FORCE, and the right shows the residuals. FA maps exhibit high similarity with minor differences along white matter tracts, while MD and RD maps show slightly increased intensity in central regions with FORCE.

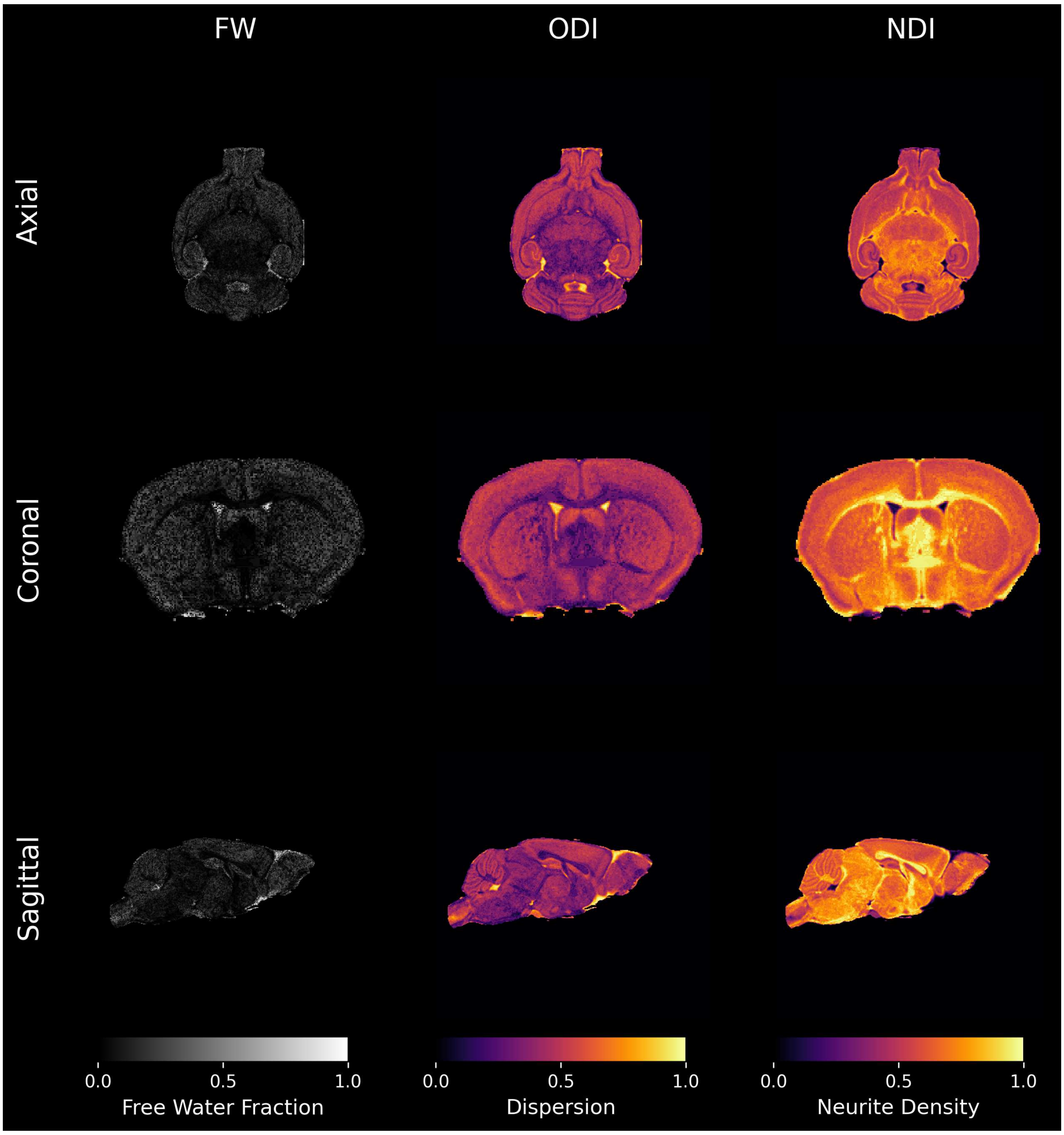

Fig. C10 NODDI parameter maps directly estimated via FORCE in single-shell ex vivo mouse data. Columns show FW fraction, ODI, and NDI maps across Axial, Sagittal, and Coronal views. Despite the single-shell acquisition, FORCE enables estimation of microstructural features, producing spatially coherent FW maps and anatomically consistent dispersion and neurite density patterns throughout gray and white matter regions. These results demonstrate the ability of FORCE to extend NODDI-like modeling to single-shell datasets, which are typically unsuitable for conventional multi-compartment fitting.

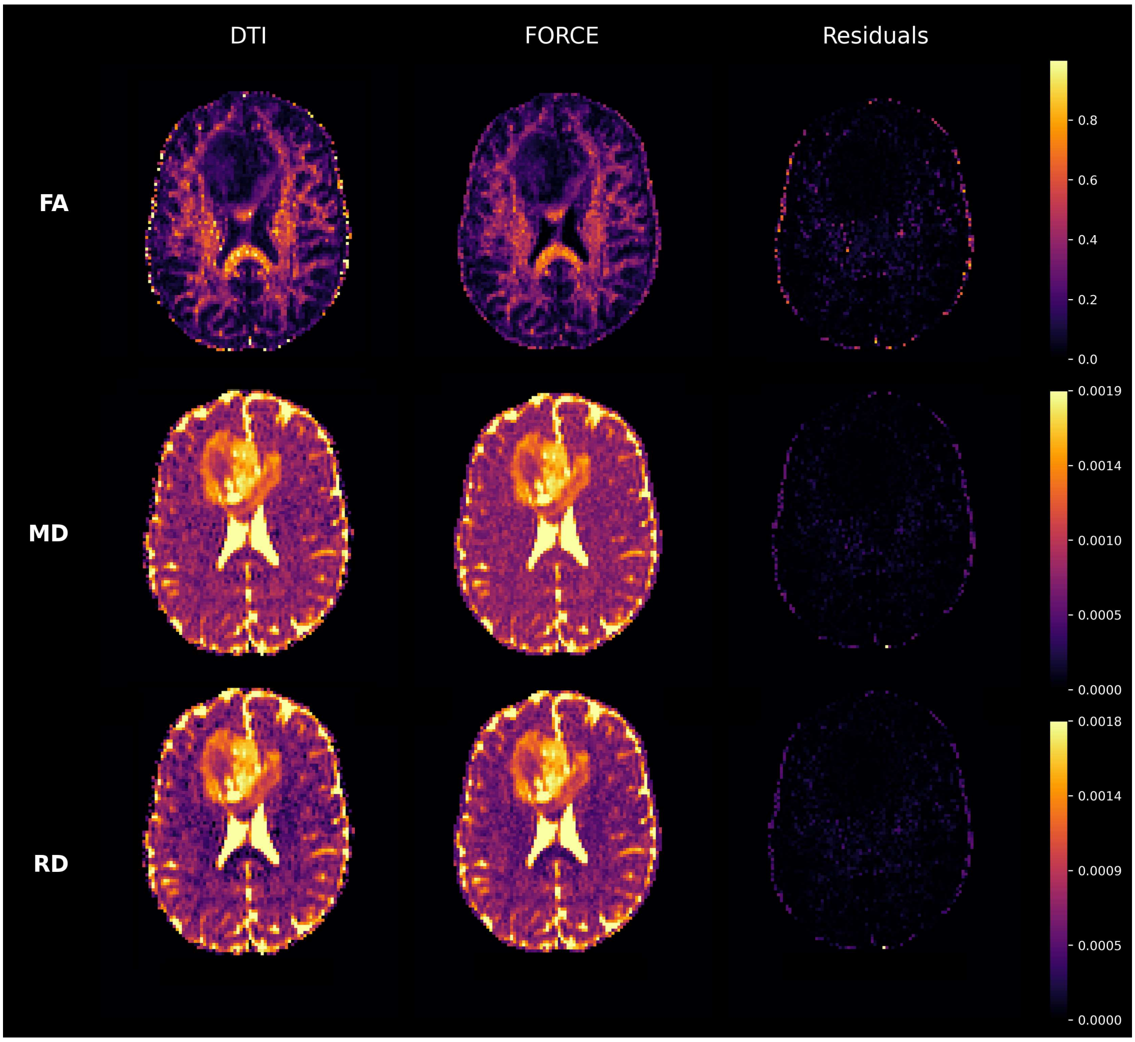

Fig. C11 The figure compares scalar maps derived from a standard DTI model (first column) and FORCE model (second column). The rows display FA, MD, and RD. The third column shows the absolute residual difference between the two models, highlighting minimal variation. The glioma is visible as an area of high signal intensity in the MD and RD maps, indicating disrupted tissue microstructure. The dataset is acquired on a single-shell of b-value 1000 for 64 gradient directions. See also Figure C12.

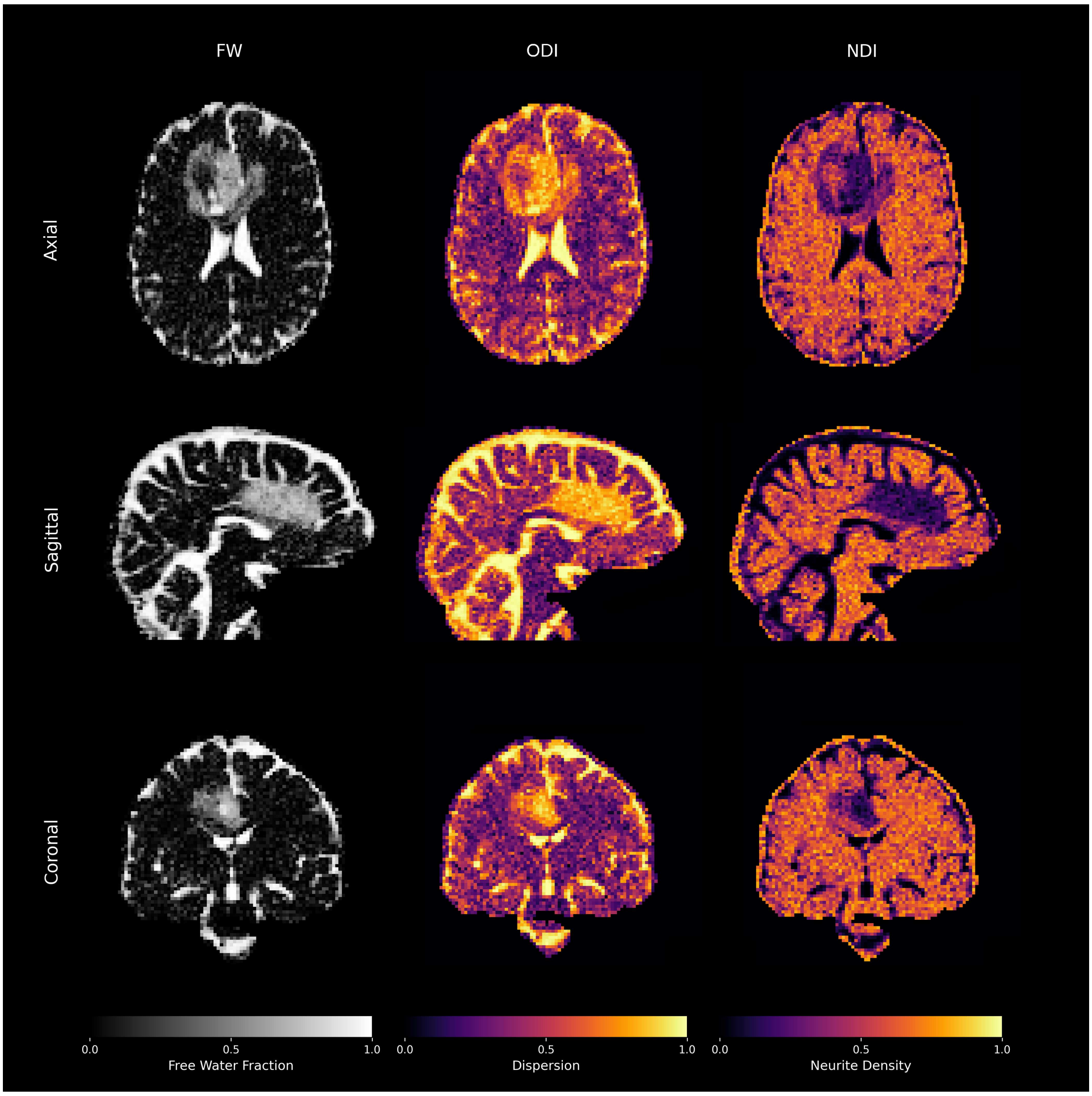

Fig. C12 This figure shows key microstructural maps from the NODDI model in three anatomical planes (Axial, Sagittal, Coronal). The columns represent the FW, ODI, and NDI. The tumor is clearly characterized by a high free water fraction and a corresponding decrease in neurite density.

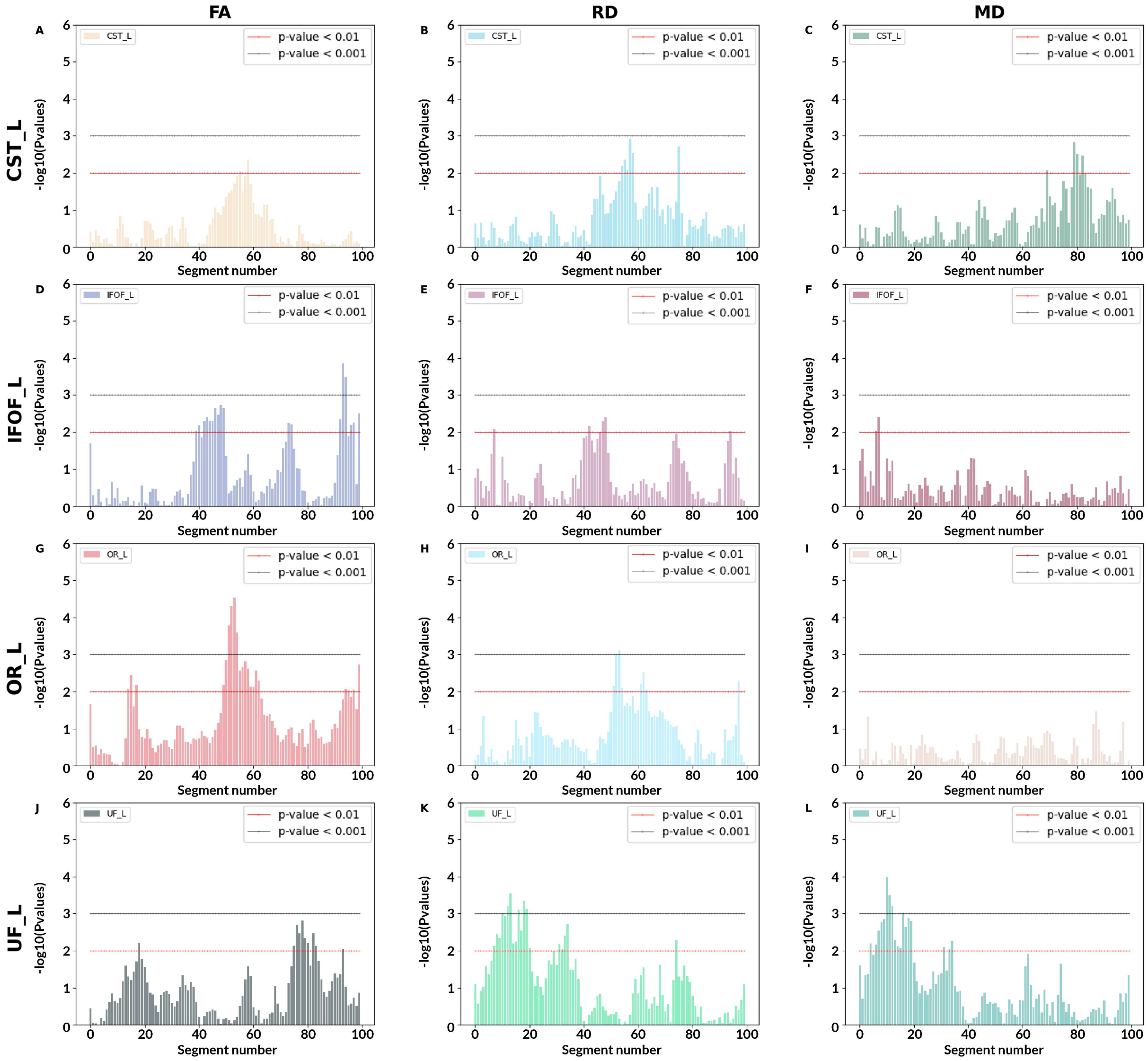

Fig. C13 BUAN pipeline analysis performed on FORCE-derived bundles and microstructure metrics. The rows display segmental significance profiles for FA (left), MD (middle), and RD (right) across four major left-hemisphere bundles. These profiles are consistent with findings from the original analyses [75] indicating that Parkinson's patients optical pathways are affected by the disease.

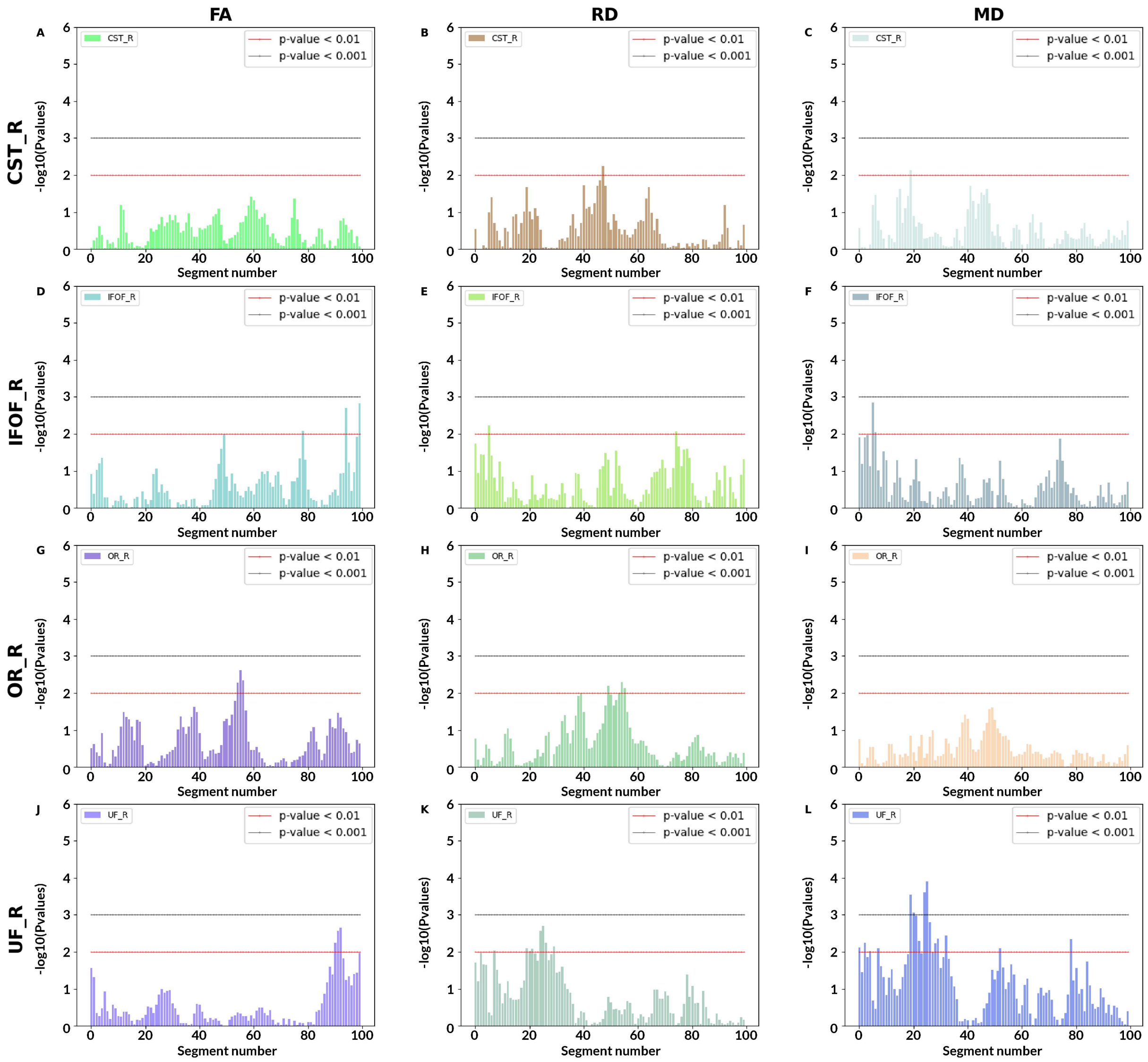

Fig. C14 BUAN pipeline analysis performed on FORCE-derived bundles and microstructure metrics. The rows display segmental significance profiles for FA (left), MD (middle), and RD (right) across four major right-hemisphere bundles. These profiles are consistent with findings from the original analyses [75] indicating that Parkinson's patients optical pathways are affected by the disease.

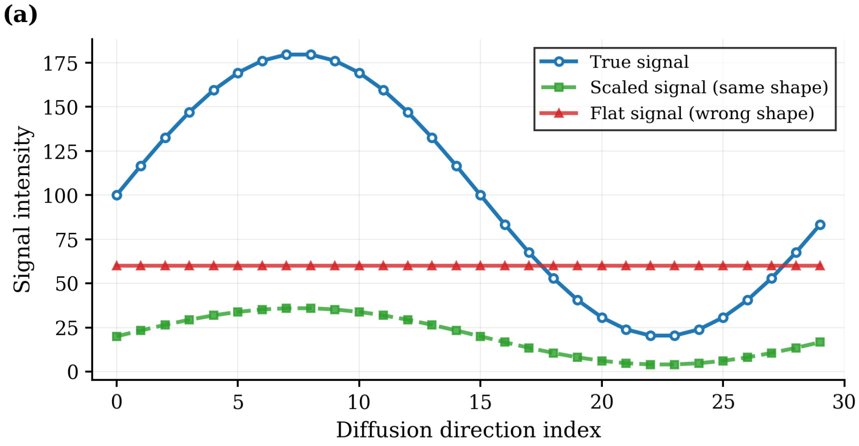

(b)

Metric	Scaled	Flat	Best Match
ℓ_2 distance	503.4	379.5	**Flat**
Cosine similarity	1.000	0.870	**Scaled**

Fig. C15 Scale invariance demonstration. (a) A true signal (blue) compared against a scaled version with identical shape (green) and a flat signal (red). (b) ℓ_2 distance incorrectly matches the flat signal due to scale differences, while cosine similarity correctly identifies the scaled signal based on shape alone.

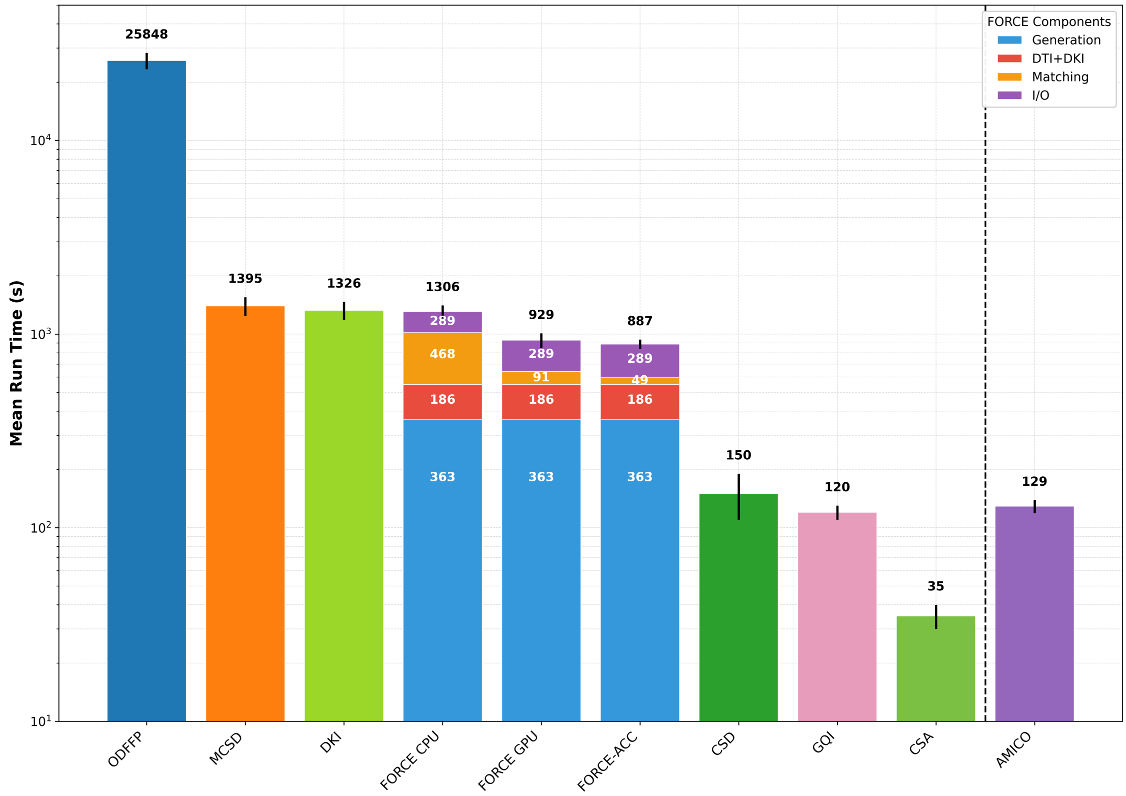

Fig. C16 Comparison of mean runtimes (in seconds, log scale) across fiber reconstruction methods. Error bars indicate variability of runtimes across 100 subjects from HCP 3T dataset. The runtimes are compared on a machine with 13th Gen Intel(R) Core(TM) i7-13700 CPU with 24 cores and RTX A6000 GPU. ODFFP and FORCE variants used 500000 simulations. All models were executed using 24 CPU cores. For FORCE, the orange bar segment denotes the per-subject matching time. Simulations were generated once and reused across subjects with identical acquisitions, so per-dataset runtime is dominated by matching and I/O rather than (re)simulation. DTI runtime is not shown because the DKI yields the DTI tensor and derived metrics as a subset

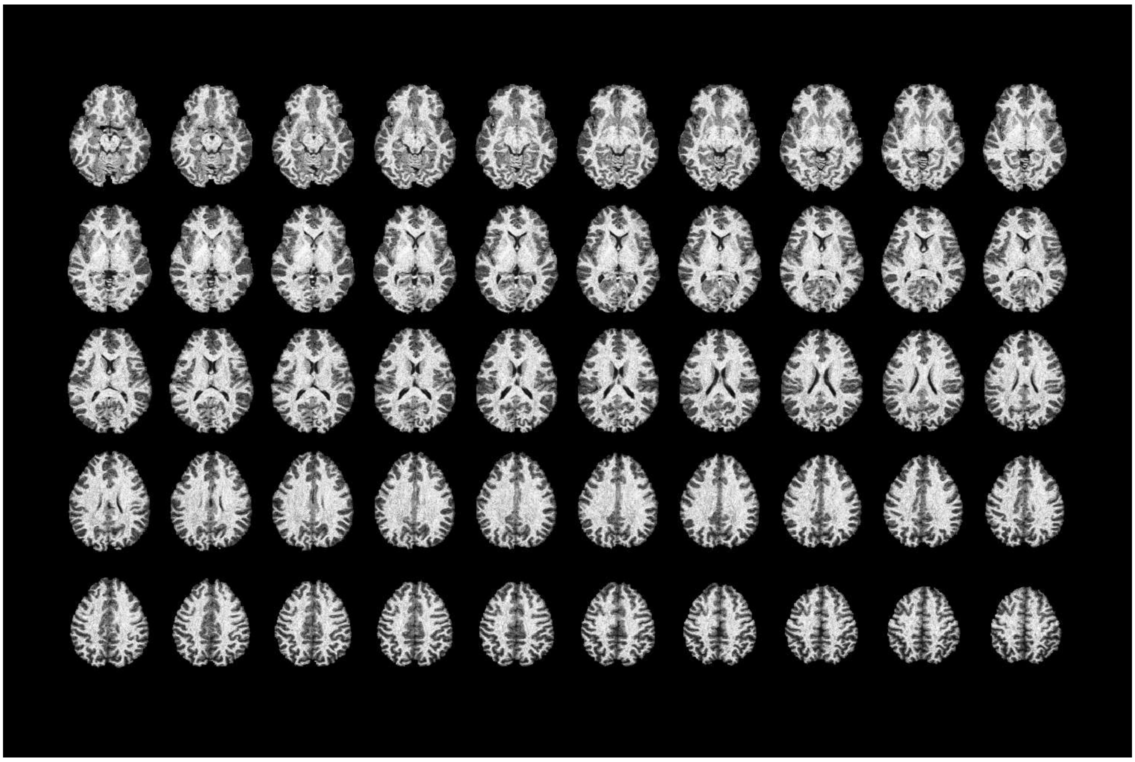

Fig. C17 WM mosaic on HCP 3T dataset subject.

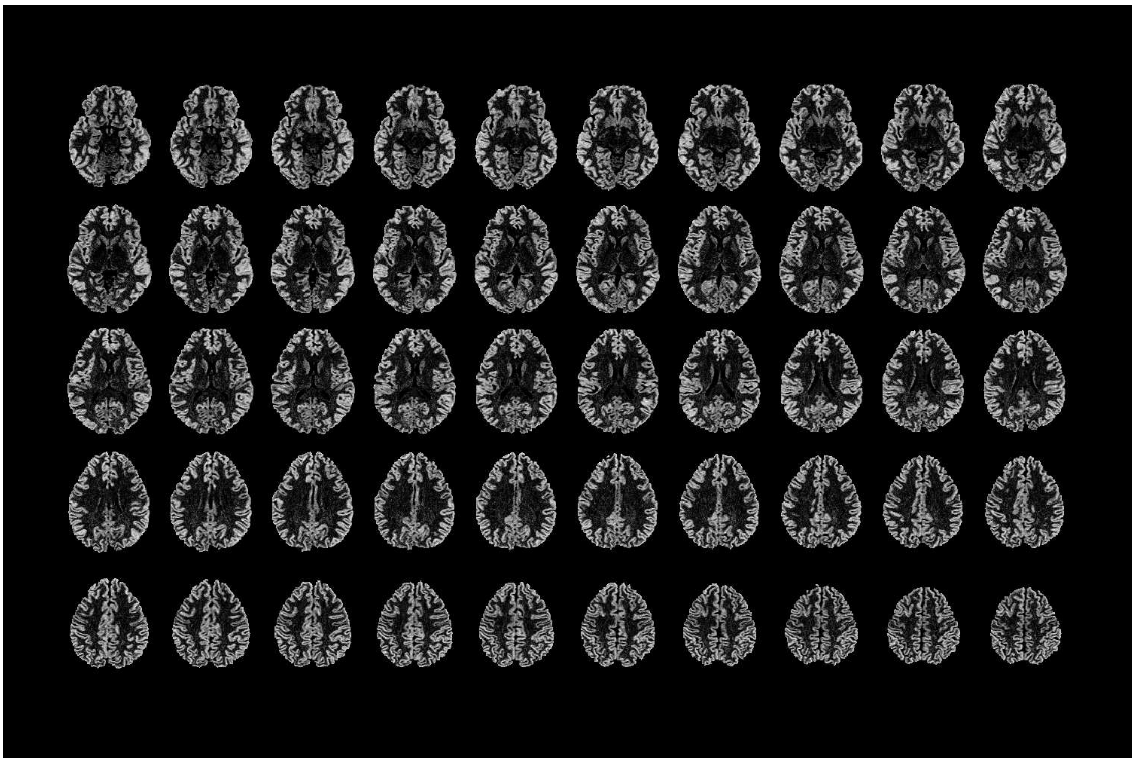

Fig. C18 GM mosaic on HCP 3T dataset subject.

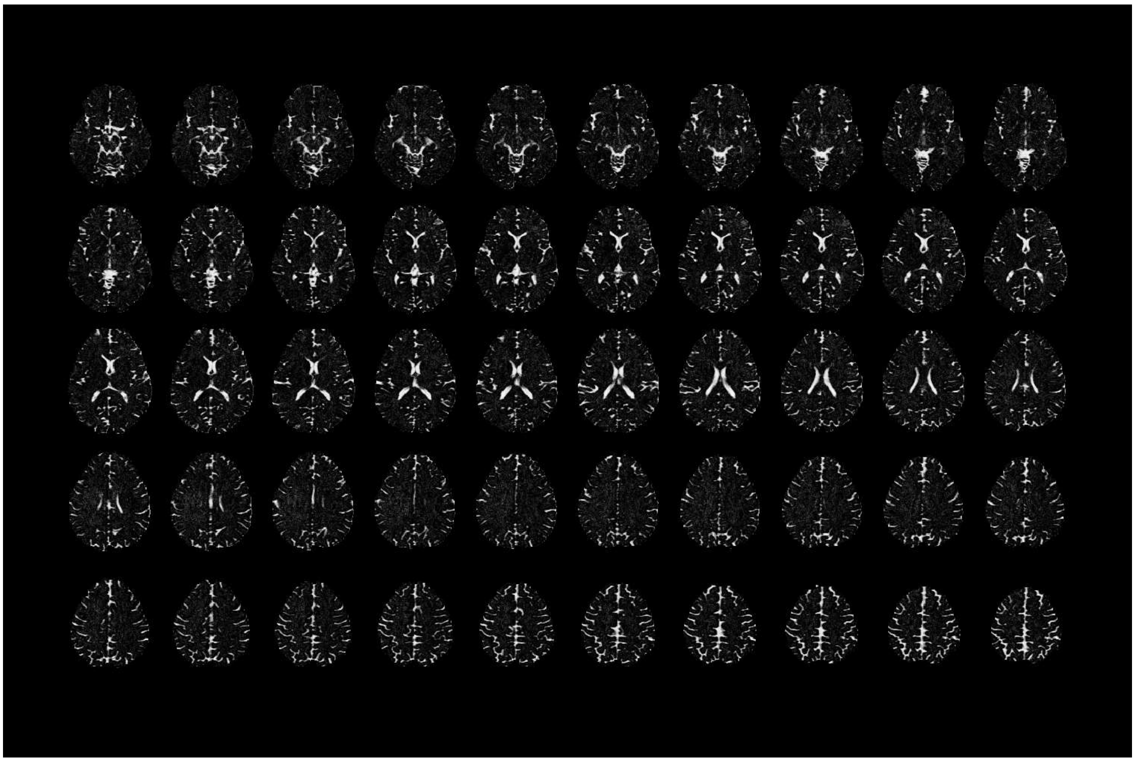

Fig. C19 FW mosaic on HCP 3T dataset subject.

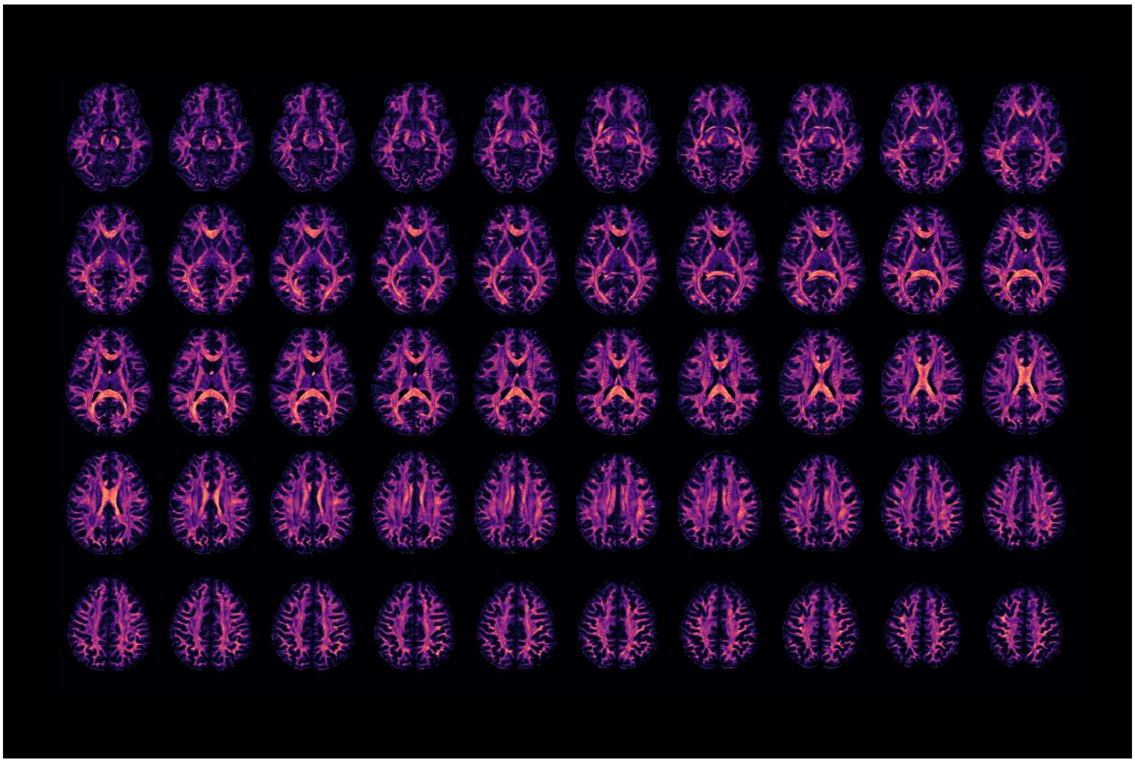

Fig. C20 FA mosaic on HCP 3T dataset subject.

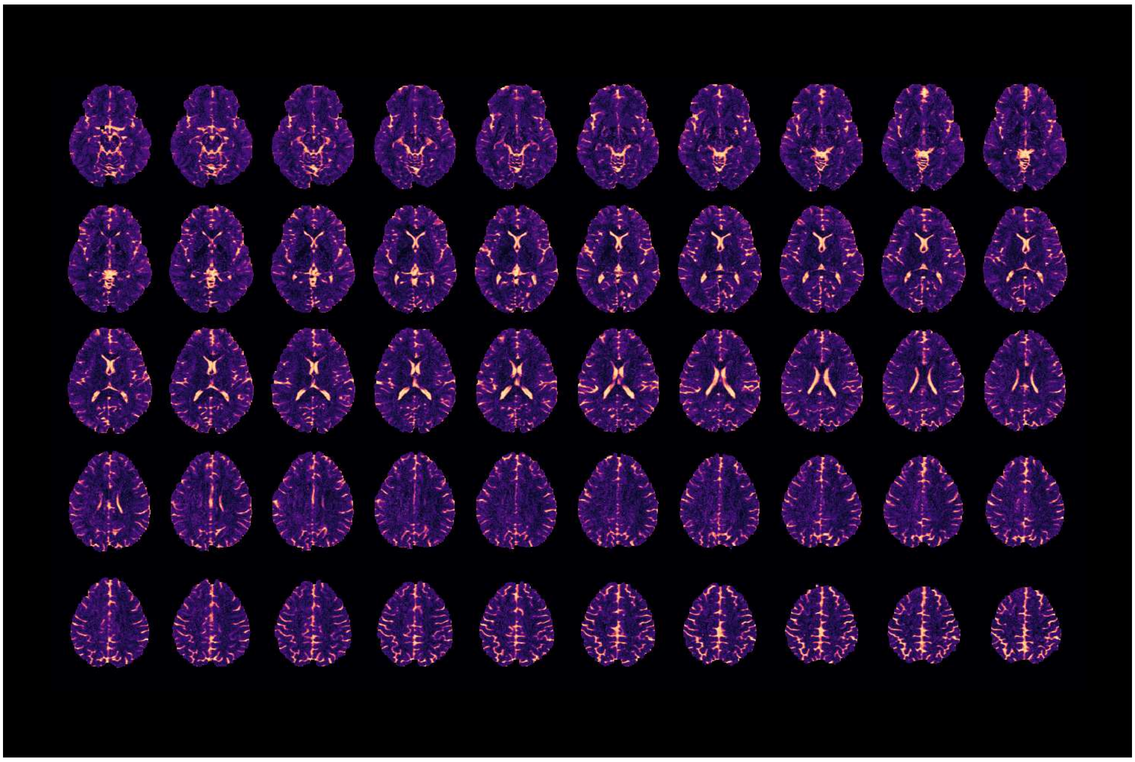

Fig. C21 RD mosaic on HCP 3T dataset subject.

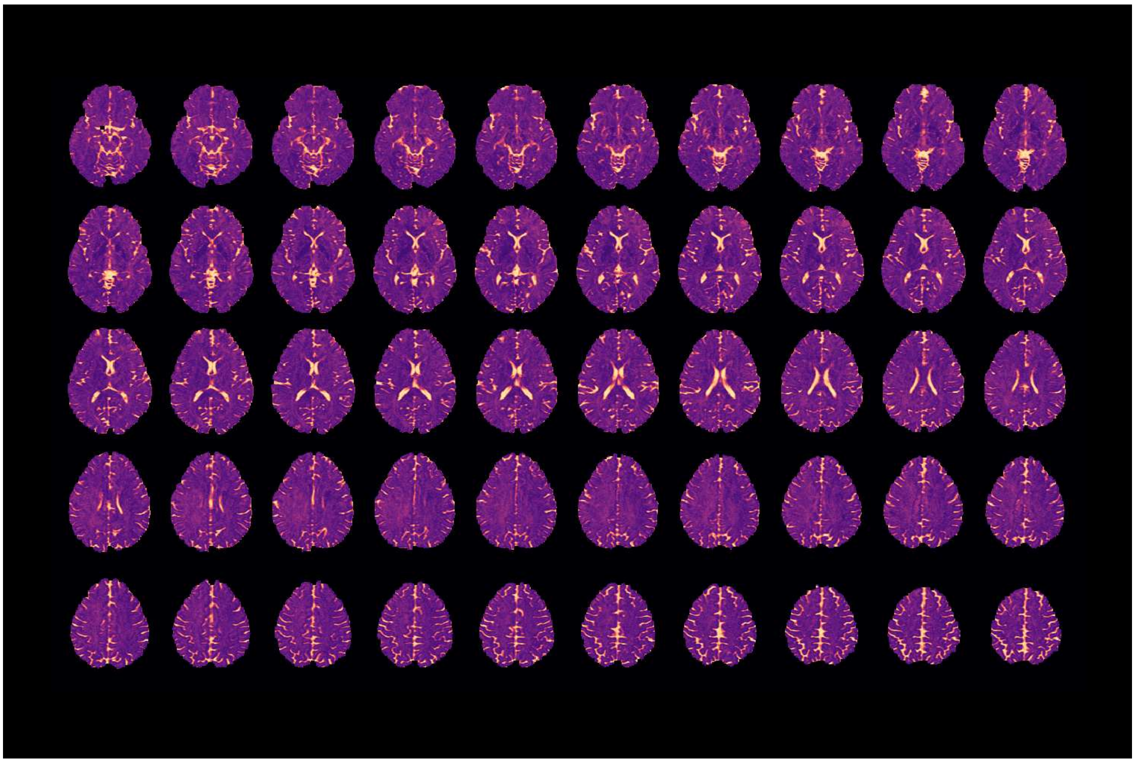

Fig. C22 MD mosaic on HCP 3T dataset subject.

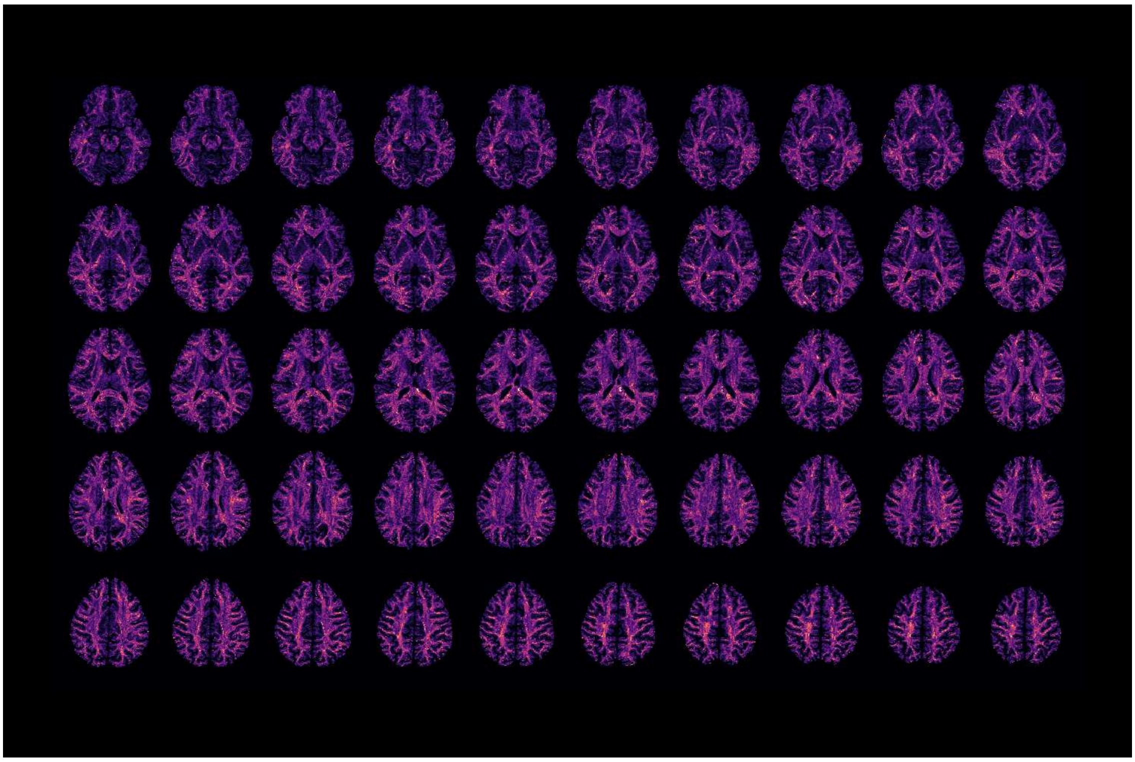

Fig. C23 KFA mosaic on HCP 3T dataset subject.

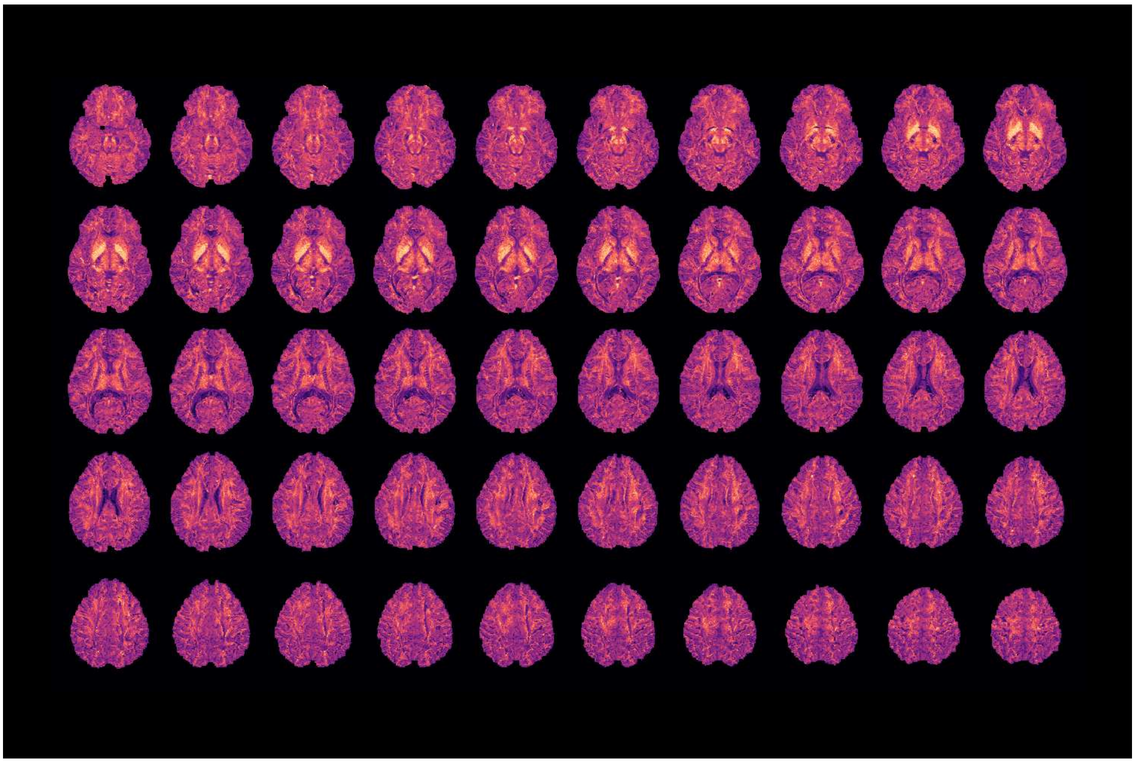

Fig. C24 AK mosaic on HCP 3T dataset subject.

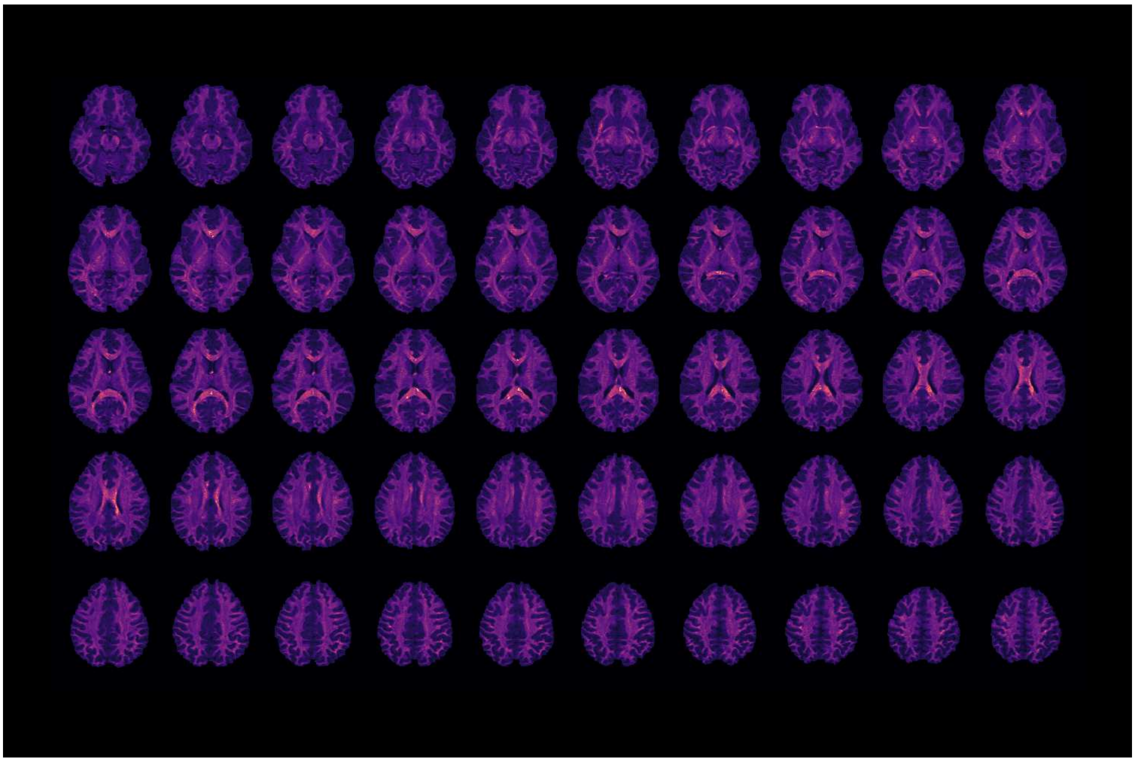

Fig. C25 RK mosaic on HCP 3T dataset subject.

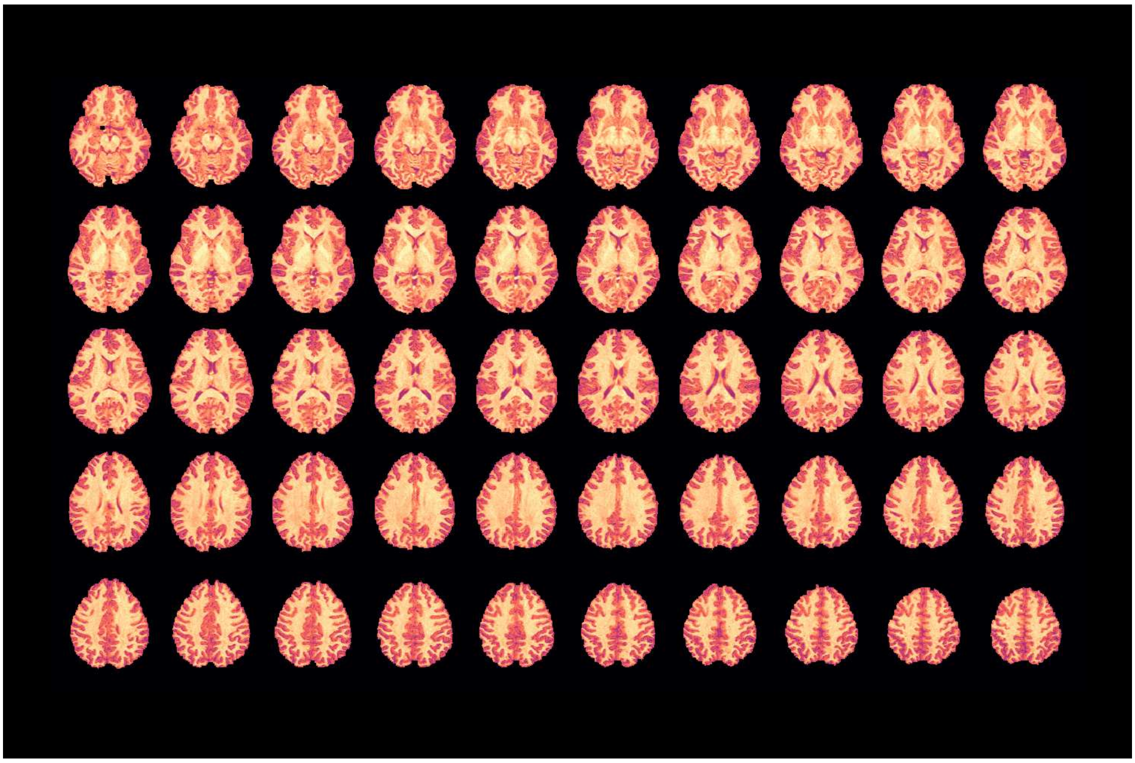

Fig. C26 micro-FA mosaic on HCP 3T dataset subject.

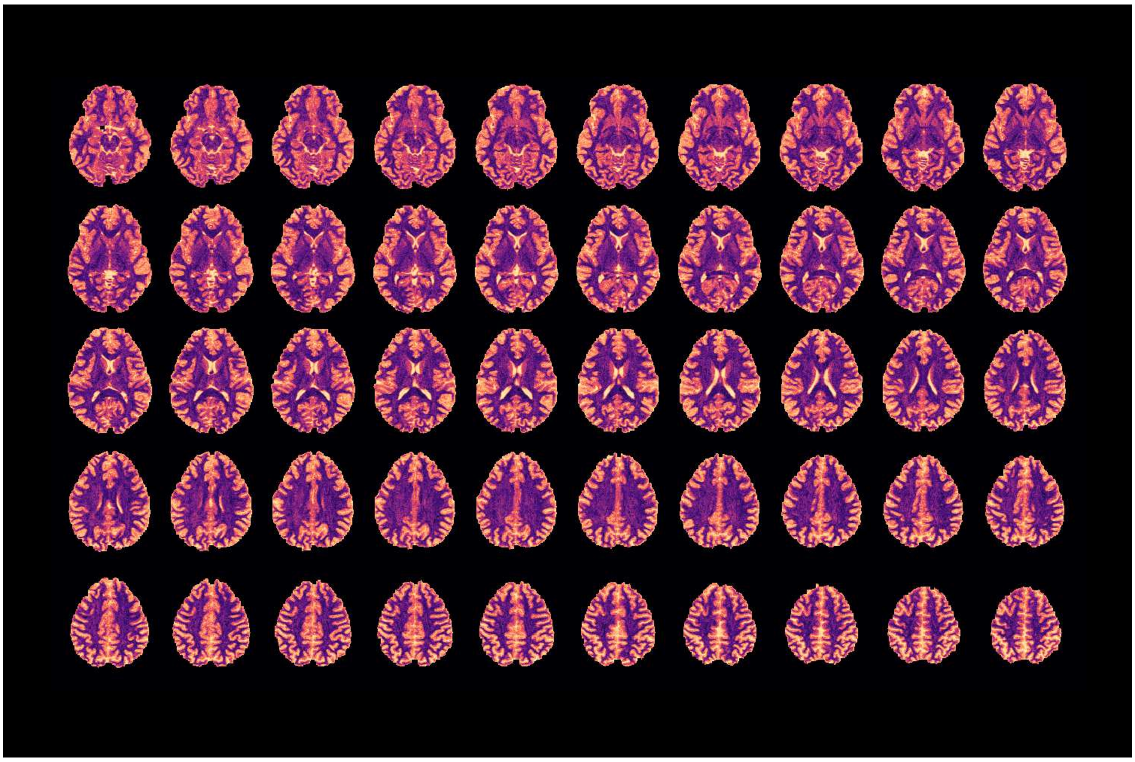

Fig. C27 ODI mosaic on HCP 3T dataset subject.

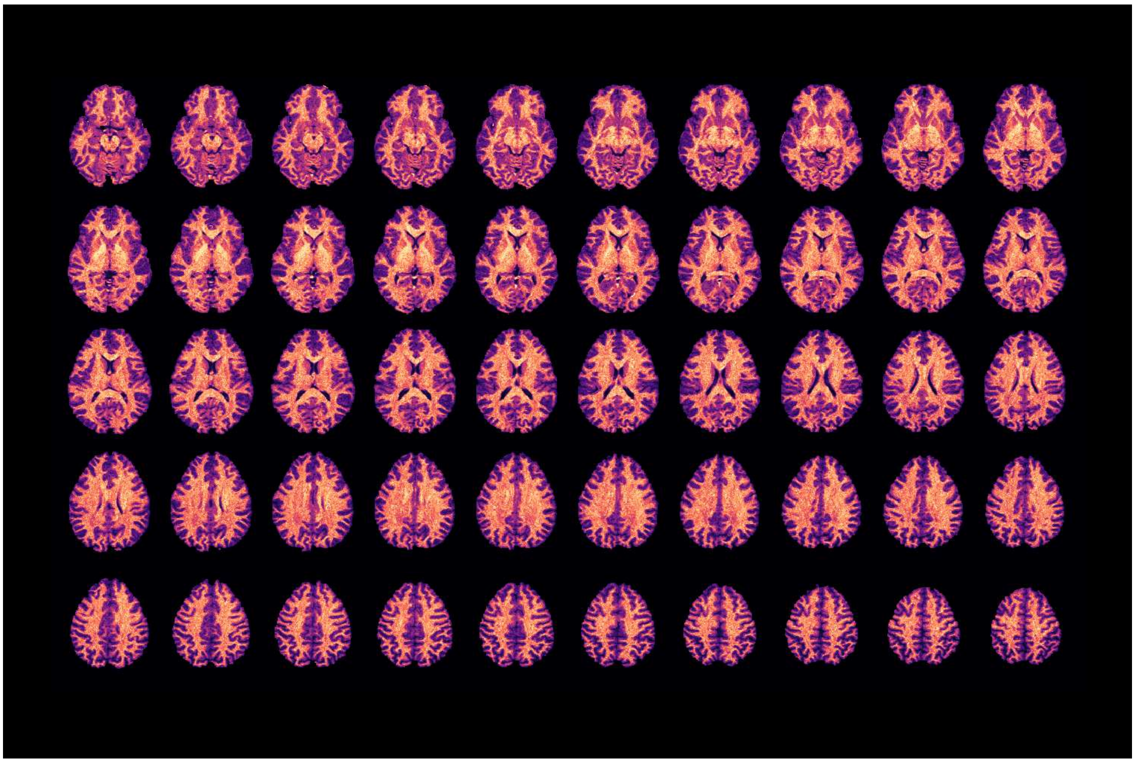

Fig. C28 NDI mosaic on HCP 3T dataset subject.

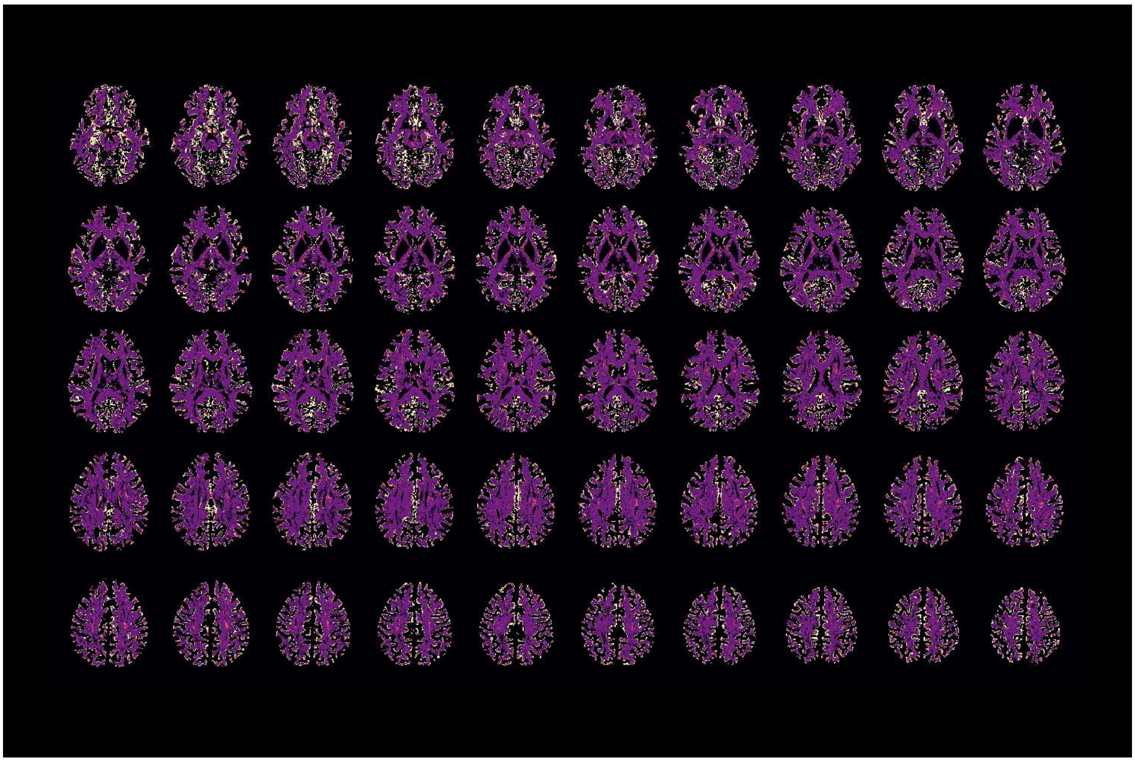

Fig. C29 Uncertainty of estimates mosaic on number of fibers on HCP 3T dataset subject.

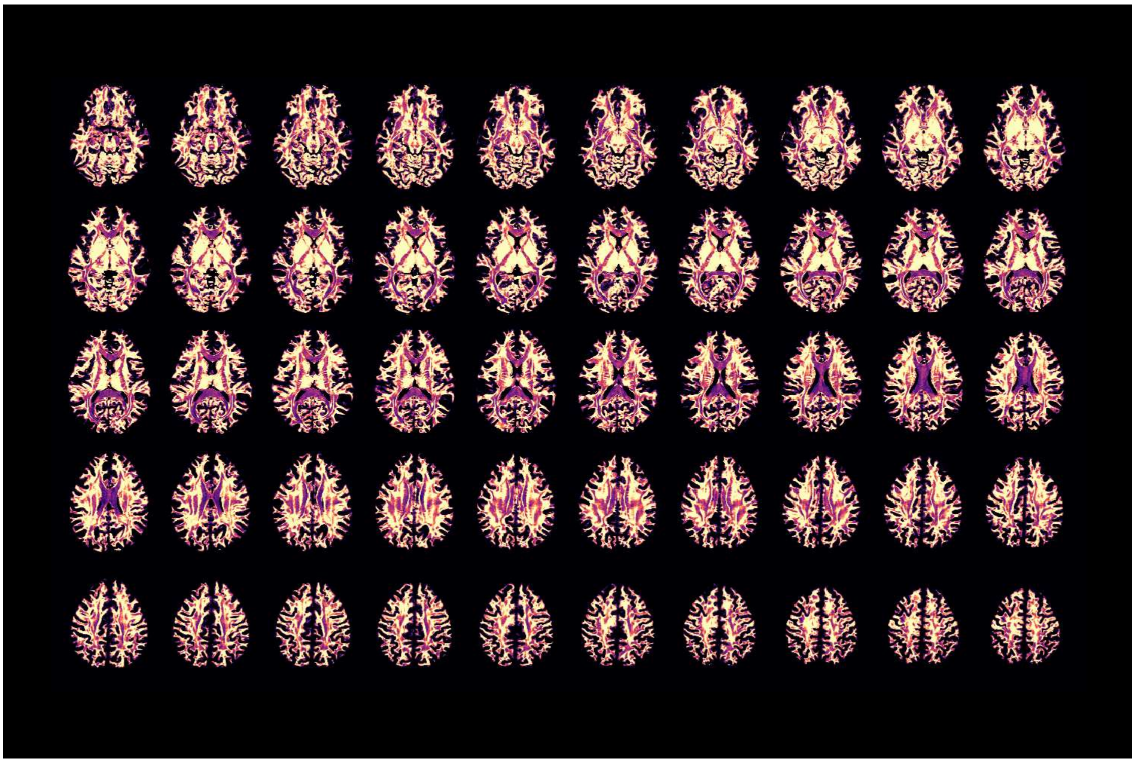

Fig. C30 Ambiguity of estimates mosaic on number of fibers on HCP 3T dataset subject.

Appendix D Summary of Contributions

1. Forward rather than inverse modeling for dMRI.
2. Framework grounded in biologically plausible biophysical models.
3. Generates a comprehensive and interpretable set of microstructural biomarkers within a single unified framework.
4. Adaptive to different dMRI acquisitions.
5. Maintains robustness across spatial resolutions.
6. Validated on both human and mice datasets.
7. Efficient and highly parallelized.
8. Accurately resolves fiber crossings at the voxel level while simultaneously producing anatomically coherent maps.
